# Club cell-specific role of programmed cell death 5 in pulmonary fibrosis

**DOI:** 10.1038/s41467-021-23277-8

**Published:** 2021-05-19

**Authors:** Soo-Yeon Park, Jung Yeon Hong, Soo Yeon Lee, Seung-Hyun Lee, Mi Jeong Kim, Soo Yeon Kim, Kyung Won Kim, Hyo Sup Shim, Moo Suk Park, Chun Geun Lee, Jack A. Elias, Myung Hyun Sohn, Ho-Geun Yoon

**Affiliations:** 1 Department of Biochemistry and Molecular Biology, Severance Medical Research Institute, Brain Korea 21 PLUS Project for Medical Sciences, Yonsei University College of Medicine, Seoul, Korea; 2 Department of Pediatrics and Institute of Allergy, Severance Medical Research Institute, Brain Korea 21 PLUS Project for Medical Sciences, Yonsei University College of Medicine, Seoul, Korea; 3 Department of Pathology, Yonsei University College of Medicine, Seoul, Korea; 4 Division of Pulmonary and Critical Care Medicine, Department of Internal Medicine, Yonsei University College of Medicine, Seoul, Korea; 5 Molecular Microbiology and Immunology, Brown University, Providence, RI USA; 6 Department of Internal Medicine, Hanyang University, Seoul, Korea

**Keywords:** Animal disease models, Respiratory tract diseases

## Abstract

Idiopathic pulmonary fibrosis (IPF) causes progressive fibrosis and worsening pulmonary function. Prognosis is poor and no effective therapies exist. We show that programmed cell death 5 (PDCD5) expression is increased in the lungs of patients with IPF and in mouse models of lung fibrosis. Lung fibrosis is significantly diminished by club cell-specific deletion of *Pdcd5* gene. PDCD5 mediates β-catenin/Smad3 complex formation, promoting TGF-β-induced transcriptional activation of matricellular genes. Club cell *Pdcd5* knockdown reduces matricellular protein secretion, inhibiting fibroblast proliferation and collagen synthesis. Here, we demonstrate the club cell-specific role of PDCD5 as a mediator of lung fibrosis and potential therapeutic target for IPF.

## Abstract (web summary)

Idiopathic pulmonary fibrosis (IPF) is a fatal adult lung disease. Here the authors investigate the functional significance of PDCD5 in club cells as a mediator of lung fibrosis and potential therapeutic target for IPF.

## Introduction

Idiopathic pulmonary fibrosis (IPF) is a lethal and progressive lung disease that affects millions of patients worldwide and causes scarring of the lungs. The cause of IPF is unknown and it is largely unaffected by current treatments^1^. Additionally, the key cellular and molecular events in early-stage IPF are poorly understood. The lungs of IPF patients display a characteristic fibrosis, which also been termed usual interstitial pneumonia. A typical interstitial pneumonia usually includes the presence of fibroblast foci, characterized by deposition of extracellular matrix (ECM), which results in distortion of normal lung structures and loss of respiratory function^2^. Since there is no effective therapy for IPF which has a mortality rate higher than many cancers, effective therapeutic targets, and treatments are urgently needed.

Club cells are non-ciliated bronchiolar epithelial cells with diverse functions including xenobiotic metabolism, immune system regulation through secretion of club cell secretory protein (CCSP), and progenitor cell activity to repair the regional epithelium in response to lung damage^3^. The role of club cells in IPF or other lung diseases is still unclear; however, there is a link between lung fibrosis and alveolar bronchiolization, a process by which club cells and other bronchiolar epithelial cell types migrate to fill the alveolar walls^4,5^. Interestingly, a recent study reported perspective on the potential pathological role of club cells in IPF, suggesting that club cells induce pulmonary epithelial cell death, leading to IPF progression^6^. In addition, club cell-specific overexpression of transforming growth factor alpha (TGF-α) activates mesenchymal cell migration and accumulation in lung fibrosis^7^. Despite the growing focus on club cells in recent years, the accumulated interest of club cells in the IPF field is very low compared with that of alveolar type II (AT2) cells. Therefore, additional studies are needed to clarify the role of club cells in the pathogenesis of pulmonary fibrosis.

Human programmed cell death 5 (*PDCD5*) is an apoptosis-associated gene located on chromosome 19q12–q13.1; the PDCD5 protein contains 125 amino acid residues. The expression of PDCD5 in various cell lines demonstrates that it has diverse functions in pathological and physiological processes^8^. PDCD5 inhibits the function of HDAC3 and promotes activation of p53 to induce apoptosis^9^. In addition, PDCD5 reduces nitric oxide production by inhibiting the physical interaction between AKT and HDAC3 in endothelial cells without affecting apoptosis^10^. Thus, PDCD5 participates in varied cellular signaling pathways via regulation of protein–protein interactions. Reduced expression of PDCD5 is common in human tumors, including gastric, breast, astrocytic glioma, chronic myelogenous leukemia, and hepatocellular carcinoma^8^. PDCD5 is also implicated in inflammatory conditions like osteoarthritis and rheumatoid arthritis, paraptosis, cell cycle regulation, ischemia/reperfusion, and immunoregulation and viral infection^11^. However, the role of PDCD5 in pulmonary fibrosis has not been explored.

In this study, we found elevated expression of PDCD5 in the lungs of patients with IPF and mouse models of lung fibrosis. Using mice with club cell-specific Pdcd5 conditional knockout (*Ccsp-Pdcd5*^*d/d*^) and AT2 cell-specific Pdcd5 conditional knockout (*Spc-Pdcd5*^*d/d*^), we show that PDCD5 plays a selective role in club cells during pulmonary fibrosis initiation. Furthermore, PDCD5 is required for formation of TGF-β-induced Smad3/β-catenin complexes on the promoter region of secreted matricellular genes in club cells. p38 MAPK phosphorylates Ser-119 of PDCD5, which enhances stability and nuclear localization of PDCD5. Finally, we verify that selective activity of PDCD5 in club cells promotes fibroblast proliferation and collagen synthesis via a paracrine loop. Our findings highlight the functional significance of PDCD5 in pulmonary fibrosis and suggest a conceptual framework for understanding the interconnectivity between club cells and pulmonary fibrosis.

## Results

### Expression of PDCD5 is elevated in the lungs of patients with IPF and in mouse models of lung fibrosis

**Fig. 1:**
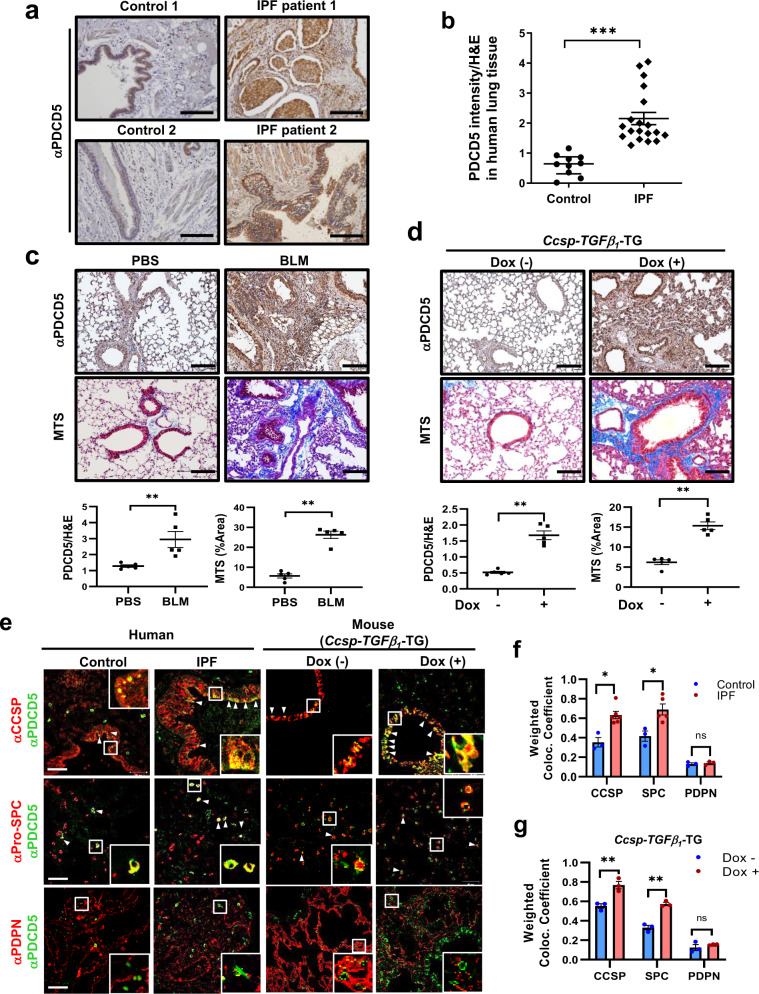
Elevated expression of PDCD5 in IPF patients and in a mouse lung fibrosis model. **a** IHC with an anti-PDCD5 antibody performed in two representative samples from control subjects and IPF patients, respectively. Scale bars = 100 μm. **b** Dot plot represents PDCD5 intensity/H&E ratio from IPF patients (*n* = 19) and the control subjects (*n* = 10). IPF vs. control; 1.7 (1.6–2.4) vs. 0.6 (0.4–0.9), *p* < 0.001, two-tailed Mann–Whitney test. Error bars, mean ± s.e.m. **c**, **d** Representative images showing PDCD5 IHC and MTS in BLM-induced fibrosis lung of wild type (**c**) and TGF-β transgenic mouse (**d**) (scale bars = 100 μm). PDCD5 and H&E intensities and MTS areas were analyzed using ImageJ software. Error bars, mean ± s.e.m. (*n* = 5 mice/group); **p < 0.008, two-tailed Mann–Whitney test (**c**, **d**). **e** Immunofluorescence assay was carried out with human and mouse lung tissue using indicated antibodies. Scale bars = 50 μm. CCSP is a club cell marker; Pro-SPC is an AT2 cell marker; and PDPN is an AT1 cell marker. Co-localized PDCD5 and each marker is indicated by white arrowheads (scale bars = 50 μm). **f**, **g** Weighted co-localized coefficient was calculated using ZEN 3.0 software. Error bars, mean ± s.e.m. *n* = 3 in each group except CCSP-IPF and SPC-IPF groups (*n* = 6). *p < 0.0357; n.s. not significant, two-tailed Mann–Whitney test (**f**). **p < 0.009; n.s. not significant, unpaired two-tailed *t-*test (**g**). Source data are provided in the Source Data file.

To investigate the pathological relevance of PDCD5 in lung disease, we examined PDCD5 expression in lung tissues from patients with IPF using immunohistochemistry (IHC). Demographic and clinical characteristics of lung tissue from control subjects and IPF patients are summarized in Supplementary Table 1. The IPF group was male dominant; age and body weights were balanced between the two groups. Lung function parameters, including forced vital capacity (FVC), forced expiratory volume in 1 s (FEV_1_), and diffusing capacity for carbon monoxide (DLCO), were significantly lower in patients with IPF compared with control (*p* < 0.001). As shown in Fig. 1a, b, PDCD5 was significantly increased in samples from IPF patients compared to those from control subjects. To further validate the expression of PDCD5 in honeycomb cysts and relatively normal airway tissue from IPF lung samples, immunofluorescence (IF) analysis was performed. Elevated levels of PDCD5 was observed in honeycomb cysts compared with normal airway tissue in IPF lungs (Supplementary Fig. 1a–c).

Bleomycin (BLM) is widely used for acute induction of lung fibrosis in mice and increases TGF-β_1_ level which is potent fibrogenic cytokine that plays a critical role in IPF pathogenesis^12^. Masson’s trichrome staining (MTS) showed that BLM induced fibrotic changes by day 14 in interstitial areas of the mouse lung. Importantly, PDCD5 levels were significantly increased in the lungs of mice following BLM injection (Fig. 1c and Supplementary Fig. 1d). TGF-β is a major inducer of epithelial–mesenchymal transition (EMT) and a key mediator of fibrosis in many tissues including the lung and kidney^13^. To further verify induction of PDCD5 expression in TGF-β-driven lung fibrosis, we next evaluated changes in PDCD5 expression in a transgenic mouse model with inducible overexpression of *TGF-β*_*1*_ (*Ccsp-TGFβ*_*1*_-TG mice), which induces lung fibrosis after administration of doxycycline (Dox). After 28 days of Dox adiministration, PDCD5 was significantly increased in the lungs of *Ccsp-TGFβ*_*1*_-TG mice compared to lungs from wild-type mice (Fig. 1d and Supplementary Fig. 1e). Bronchial lavage (BAL) fluid cell counting revealed that inflammatory cells were induced in the severe lung fibrosis mouse models (Supplementary Fig. 2a, b). Using co-IF analysis, we found that expression of PDCD5 was significantly increased in patients with IPF in club cells and AT2 cells, but not in AT1 cells (Fig. 1e left panel, f). We observed similar expression patterns in both *Ccsp-TGFβ*_*1*_-TG mice (Fig. 1e right panel, g) and BLM-induced fibrosis model mice (Supplementary Fig. 2c).

To further evaluate the co-expression of PDCD5 with other epithelial cell makers in IPF patients, co-IF staining was performed using Muc5AC (a goblet cell marker), Foxj1 (a ciliated cell marker) antibodies, and a Scgb3a2 antibody, which is another club cell marker. Similar to the results obtained from the IF analysis using CCSP, which showed the co-localization of PDCD5 with another club cell marker, the co-expression between PDCD5 and Scgb3a2 was significantly increased in IPF patients, but PDCD5 co-localization did not increase significantly with goblet cell or ciliated cell markers. Intriguingly, we also observed that PDCD5 was highly co-localized with a fibroblast marker, α-smooth muscle actin (α-SMA) in IPF patients, suggesting a plausible role for PDCD5 in myofibroblast activation during IPF development (Supplementary Fig. 2d). These results collectively demonstrate that PDCD5 expression increases in IPF and mouse models of lung fibrosis.

### Lung fibrosis is regulated by PDCD5 in a lung club cell-specific manner

To determine the physiological role of PDCD5 in the development of lung fibrosis, we generated a genetically engineered mouse model with tamoxifen-inducible *Pdcd5* ablation in lung epithelial club cells^14–16^ (*Ccsp-Pdcd5*^*d/d*^) or AT2 cells^17^ (*Spc-Pdcd5*^*d/d*^). First, to verify *Cre* activity in club cells and AT2 cells after 4-OHT treatment which is a metabolite of the tamoxifen, we adapted a dual fluorescent membrane-localized tdTomato/eGFP (mTmG) indicator mouse model, which marks *Cre*-mediated excision via a heritable switch from tdTomato expression to eGFP expression^18^. We crossed this *Rosa26-mTmG* mouse with either *Ccsp-Pdcd5*^*d/d*^ or *Spc-Pdcd5*^*d/d*^ mice to visualize the *Cre*-mediated excision of *Pdcd5* in club cells or AT2 cells, respectively. The bronchiolar epithelia of the resulting *Ccsp-Pdcd5*^*d/d*^*;mTmG* mice displayed bright eGFP expression, whereas the bronchiolar epithelia of *Pdcd5*^*f/f*^*;mTmG* mice lacked eGFP expression (Supplementary Fig. 3a). Additionally, eGFP^+^ cells were expressed in the alveolar area of *Spc-Pdcd5*^*d/d*^*;mTmG* mouse lungs, whereas *Pdcd5*^*f/f*^*;mTmG* mice lacked eGFP signal in the alveolar region (Supplementary Fig. 3b). Increased eGFP intensity was observed in the airways of *Ccsp-Pdcd5*^*d/d*^*;mTmG* mice after 4-OHT treatment. In contrast, 4-OHT-induced eGFP signal was observed in the parenchymal region of *Spc-Pdcd5*^*d/d*^*;mTmG* mice. To verify *Pdcd5* ablation in the respective lung epithelial cells from *Ccsp-Pdcd5*^*d/d*^ and *Spc-Pdcd5*^*d/d*^ mouse lungs, PDCD5 expression was verified by co-IF analysis with cell type-specific markers (Supplementary Fig. 4a, b). Furthermore, the deletion of *Pdcd5* was confirmed by quantitative reverse transcription-PCR (qRT-PCR) in isolated primary club cells and AT2 cells, which were obtained from each knockout mouse using fluorescence-activated cell sorting (FACS, Supplementary Fig. 4c–f).

**Fig. 2:**
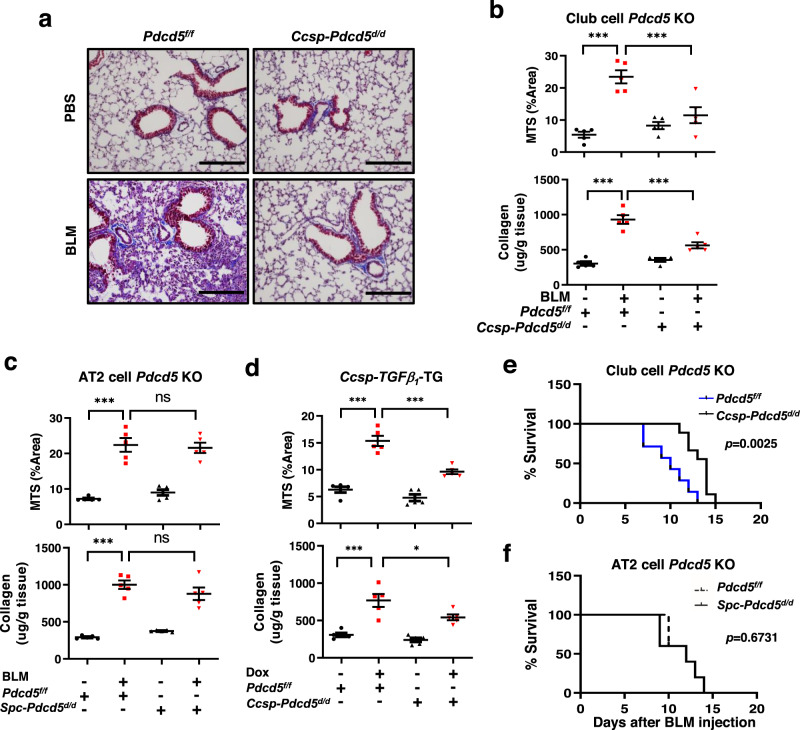
Club cell-specific deletion of *Pdcd5* prevents lung fibrosis. **a** MTS was carried out on lung tissues from *Pdcd5*^*f/f*^ and *Ccsp-Pdcd5*^*d/d*^ mice with or without BLM treatment (scale bars = 200 μm). **b**–**d** MTS quantification and soluble collagen assay using lung tissues from *Pdcd5*^*f/f*^ and *Ccsp-Pdcd5*^*d/d*^ mice (**b**), *Pdcd5*^*f/f*^ and *Spc-Pdcd5*^*d/d*^ mice (**c**), and *Ccsp-TGFβ*_*1*_-TG (**d**), respectively (*n* = 5 mice/group). Error bars represents mean ± s.e.m. Statistical analysis was performed with ANOVA with Tukey’s test. ***p < 0.001; *p = 0.029 (**b**–**d**). **e** Kaplan–Meier survival analysis was performed after BLM injection. *Pdcd5*^*f/f*^ (*n* = 7 mice) and *Ccsp-Pdcd5*^*d/d*^ (*n* = 8 mice)*, p* = 0.0025. **f** Kaplan–Meier survival of *Pdcd5*^*f/f*^ and *Spc-Pdcd5*^*d/d*^ (*n* = 5 mice/group), *p* = 0.6731. Statistical analysis was performed using log-rank (Mantel–Cox) test (**e, f**). Source data are provided in the Source Data file.

Next, we induced lung fibrosis in these mouse models using BLM injection through the trachea. We found that BLM-induced lung fibrosis was markedly diminished in *Ccsp-Pdcd5*^*d/d*^ mice, quantified using MTS-stained areas in the lung and soluble collagen content via Sircol Collagen Assay (Fig. 2a, b). In contrast, there were no significant changes related to fibrosis and collagen synthesis in *Spc-Pdcd5*^*d/d*^ mice (Fig. 2c and Supplementary Fig. 5a). To further examine the role of PDCD5 in club cell-specific lung fibrosis, inducible *Ccsp-TGFβ*_*1*_-TG mice were bred to *Ccsp-Pdcd5*^*d/d*^ mice to generate ablation of *Pdcd5* and overexpression of *TGF-β*_*1*_ in the club cells (Supplementary Fig. 5b). Following administration of Dox, wild-type mice developed lung fibrosis; however, previously observed increased lung fibrosis was significantly diminished in *Ccsp-Pdcd5*^*d/d*^ mice (Fig. 2d and Supplementary Fig. 5c). Moreover, we compared the survival rate after BLM injection in both club cell- and AT2 cell-specific *Pdcd5* knockout mice. Kaplan–Meier survival analysis demonstrated there was prolonged survival in *Ccsp-Pdcd5*^*d/d*^ mice (Fig. 2e), whereas there was no significant survival change in *Spc-Pdcd5*^*d/d*^ mice (Fig. 2f). Importantly, club cell-specific knock-out of Pdcd5 gene had no effects on induction of PDCD5 expression by BLM in both AT2 cells and fibroblasts (Supplementary Fig. 6). These data suggested that PDCD5 in the club cells plays an important role in the initiation of lung fibrosis.

To further validate the functional role played by PDCD5 in the initiation of pulmonary fibrosis, we performed the genetic deletion of *Pdcd5* after the induction of lung fibrosis. We first examined the time course of lung fibrosis induction following BLM injection. We found that BLM significantly induced lung fibrosis and PDCD5 expression starting 3 days after injection (Supplementary Fig. 7a). Thus, *Ccsp-Pdcd5*^*d/d*^ mice were treated with 4-OHT, 3 days after BLM injection. As shown in Supplementary Fig. 7b, the induction of lung fibrosis following BLM injection was significantly suppressed by deletion of *Pdcd5* gene from 2 days after first 4-OHT injection. These data revealed PDCD5 mediates lung fibrosis initiation. It was also noteworthy that *Pdcd5* depletion did not affect cell death of lung (Supplementary Fig. 8a). Furthermore, we examined the effects of *Pdcd5* deletion on the proliferation of club cells through IF analysis, using antibodies against Ki67 and CCSP (Supplementary Fig. 8b). *Pdcd5* ablation did not appear to affect the proliferation of club cells. Taken together, our results suggest that PDCD5 mediates lung fibrosis initiation, without affecting club cell death and proliferation.

### PDCD5 promotes TGF-β signaling by mediating formation of a Smad3/PDCD5/β-catenin complex

**Fig. 3:**
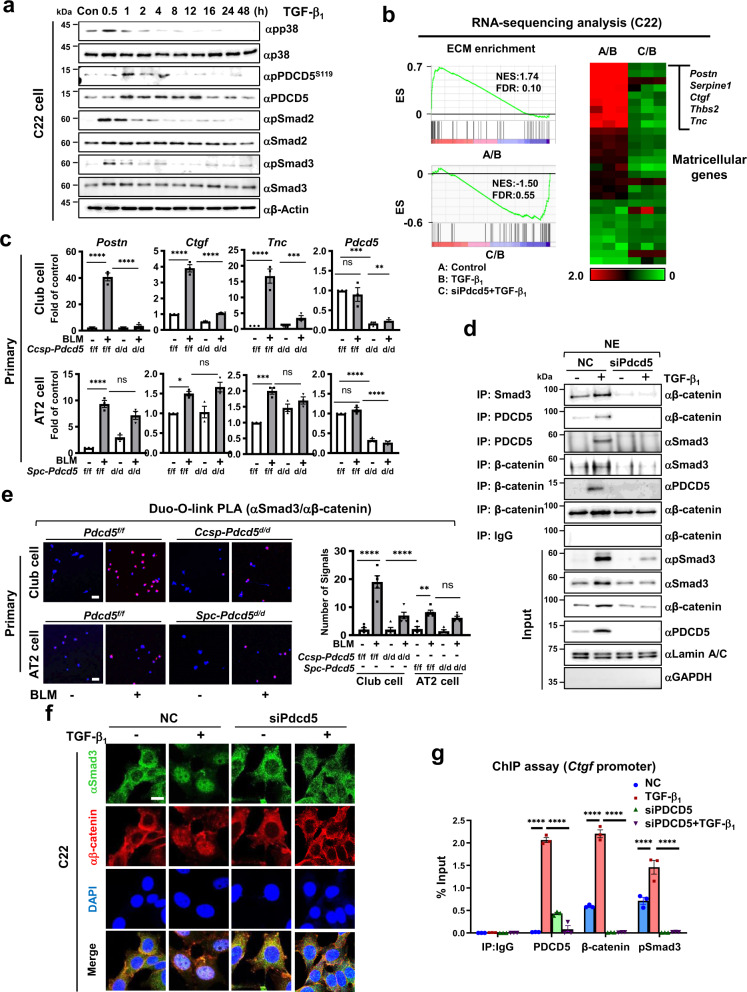
PDCD5 mediates TGF-β-dependent transcriptional activation of matricellular genes via formation of a PDCD5/β-catenin/Smad3 complex. **a** C22 cells were treated with TGF-β_1_ (20 ng/ml) and harvested at indicated time points. Whole-cell lysates were analyzed by immunoblotting with indicated antibodies. Representative blots for three independent experiments are shown. **b** GSEA of RNA-seq signals of GO-defined ECM gene clusters in C22 cells. The normalized enrichment score (NES) is pictured in the left panel. A heat map of the ECM gene signature is pictured in the right panel. **c** Primary club cells were isolated from *Pdcd5*^*f/f*^ and *Ccsp-Pdcd5*^*d/d*^ mouse lungs, and AT2 cells were isolated from *Pdcd5*^*f/f*^ and *Spc-Pdcd5*^*d/d*^ mice after BLM injection. The levels of indicated genes were analyzed by qRT-PCR. (*n* = 3 mice/group); *p = 0.0293; **p = 0.0034; ***p < 0.0008; ****p < 0.0001; n.s. not significant. **d** C22 cells were transfected with *Pdcd5* siRNAs for 36 h and then treated with TGF-β_1_ for 12 h. Nuclear extracts were immunoprecipitated with the indicated antibodies and immunoblotted with the indicated antibodies. Representative blots from three independent experiments are shown. **e** Primary club cells or AT2 cells from BLM-injected mice were analyzed by in situ PLA, using Smad3 and β-catenin antibodies. Scale bars = 20 µm. Number of signals was quantified using ImageJ software (*n* = 5/group); **p = 0.0074; ****p < 0.0001; n.s. not significant. **f** C22 cells were treated with *Pdcd5* siRNA for 36 h and/or TGF-β_1_ for 12 h, immunofluorescence analysis was performed. Scale bars = 10 μm. Representative images from three independent experiments are shown. **g** C22 cells were treated with TGF-β_1_ for 24 h and a ChIP assay was performed using the indicated antibodies. Precipitated samples were analyzed by qRT-PCR, and results are presented as the percentage of input (*n* = 3 in each group); ****p < 0.0001. Data are shown as mean ± s.e.m. Statistical analysis was performed with one-way ANOVA with Tukey’s post hoc test (**c**, **e**, **g**). Source data are provided in the Source Data file.

We next examined the molecular mechanism by which PDCD5 mediates lung fibrosis in club cells. To do this, we used conditionally immortalized C22 mouse club cells. We previously showed that in response to genotoxic stress, levels of PDCD5 and phosphorylated PDCD5 concurrently increase in HCT-116 cells^9,19^. We thus examined whether TGF-β_1_ treatment changes PDCD5 expression and/or phosphorylation in C22 cells. After 1 h of TGF-β_1_ treatment, both total and phosphorylated levels of PDCD5 were increased, and PDCD5 expression was continuously stimulated until 12 h post-treatment. As expected, both Smad2 and Smad3 phosphorylation were also induced early following TGF-β treatment in C22 cells (Fig. 3a). However, no significant change in PDCD5 phosphorylation and expression was observed following TGF-β_1_ treatment in the RLE-6TN AT2 cell line (Supplementary Fig. 9a).

To determine whether PDCD5 is involved in transcriptional regulation of TGF-β target genes, we performed RNA sequencing (RNA-seq) analysis in C22 club cells with or without knocking-down of *Pdcd5* after 24 h of TGF-β_1_ treatment. Gene set enrichment analysis (GSEA) demonstrated significant enrichment of ECM genes in TGF-β_1_-treated C22 cells. However, *Pdcd5* knockdown negatively regulated ECM Gene Ontology (GO)-defined genes (Fig. 3b). Among TGF-β-regulated ECM genes, the top 10 included matricellular genes such as periostin (*Postn*), plasminogen activator inhibitor-1 (*Serpine1*), connective tissue growth factor (*Ctgf*), thrombospondin 2 (*Thbs2*), and tenascin C (*Tnc*); these genes were significantly down-regulated following *Pdcd5* knockdown in TGF-β_1_-treated C22 cells. Additionally, we verified our RNA-seq data with quantitative RT-PCR (Supplementary Fig. 9b). In contrast to our findings in C22 cells, *Pdcd5* knockdown had no effects on the mRNA levels of these matricellular genes in AT2 cells (Supplementary Fig. 9c). These findings were again verified using primary club cells and AT2 cells from *Ccsp-Pdcd5*^*d/d*^ and *Spc-Pdcd5*^*d/d*^ mouse lungs (Fig. 3c). Collectively, these findings suggest that PDCD5 selectively regulates transcription of pro-fibrotic matricellular genes in the club cells.

β-Catenin interacts with Smad3 on TGF-β target gene promoters in lung epithelial cell lines, leading to EMT and fibrosis^20^. Since PDCD5 acts as a molecular chaperone by mediating protein–protein interaction^8^, we next examined whether PDCD5 interacts with β-catenin or Smad3. Co-immunoprecipitation analysis showed that endogenous PDCD5 interacts with β-catenin and Smad3 in the nucleus; these interactions were further enhanced following treatment with TGF-β_1_ in C22 cells (Supplementary Fig. 10a). We also verified the selective interaction of PDCD5 with Smad3 but not with other Smad proteins (Supplementary Fig. 10b). An in vitro binding assay showed that PDCD5 directly interacts with β-catenin, but not Smad3 (Supplementary Fig. 10c). We next tested whether PDCD5 is required for formation of the Smad3/β-catenin complex in a response to TGF-β stimuli. Following *Pdcd5* knockdown, TGF-β-induced Smad3/β-catenin interaction was abolished in the nucleus in C22 cells (Fig. 3d). An in situ proximal ligation assay also verified that the TGF-β-induced Smad3/β-catenin interaction occurred in the nucleus and that this signal was reduced following the *Pdcd5* knockdown in C22 club cells (Supplementary Fig. 10d). Furthermore, the level of BLM-induced Smad3/β-catenin interaction was significantly diminished by *Pdcd5* deletion in primary club cells but not in AT2 cells (Fig. 3e and Supplementary Fig. 10e). As reported previously, both Smad3 and β-catenin were translocated into the nucleus upon TGF-β_1_ treatment^20^; however, *Pdcd5* knockdown significantly reduced TGF-β-induced nuclear translocation of both Smad3 and β-catenin (Fig. 3f and Supplementary Fig. 10f). Because the Smad3/β-catenin complex binds to the promoter region of TGF-β target genes, such as *Ctgf*, we tested whether PDCD5 is also recruited to same Smad-binding element in the *Ctgf* gene. Chromatin immunoprecipitation (ChIP) analysis showed that the PDCD5/β-catenin/Smad3 complex is recruited to the Smad-binding element of *Ctgf* gene in a response to TGF-β_1_ treatment in C22 cell line. Importantly, this recruitment was abolished following *Pdcd5* knockdown (Fig. 3g). We observed similar effect on another matricellular gene, *Postn* (Supplementary Fig. 10g). These results indicate that PDCD5 promotes TGF-β-induced transcriptional activation of matricellular genes by mediating formation of the PDCD5/β-catenin/Smad3 complex.

### p38 MAPK phosphorylates PDCD5 Ser-119, leading to PDCD5 stabilization and nuclear translocation in a response to TGF-β

**Fig. 4:**
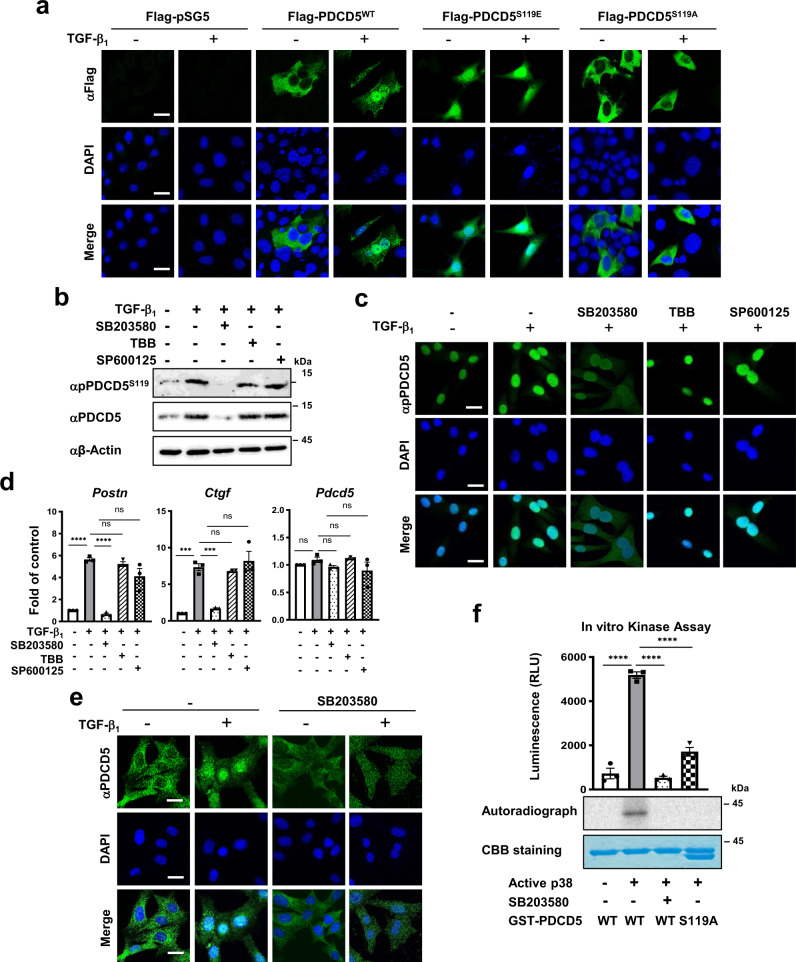
p38 MAPK promotes stabilization and nuclear translocation of PDCD5 in a response to TGF-β. **a** C22 cells were transfected with the indicated plasmids for 24 h and then treated with TGF-β_1_ for 12 h. Immunofluorescence staining was performed with a FITC-conjugated Flag antibody. Scale bars = 50 μm. **b** C22 cells were treated with each of the indicated inhibitors (10 μM SB203580; 10 μM TBB; and 10 μM SP600125) for 2 h before TGF-β_1_ (20 ng/ml) treatment. Immunoblotting with the indicated antibodies was then carried out. Representative blots from three independent experiments are shown. **c**, **e** C22 cells were treated with TGF-β_1_ and/or indicated inhibitors. Immunofluorescence staining was performed with anti-p-PDCD5 and anti-PDCD5 antibodies after 12 h TGF-β_1_ treatment. Scale bars = 50 μm. **d** C22 cells were treated with TGF-β_1_ and/or indicated inhibitors. The levels of indicated genes were analyzed by qRT-PCR. Error bars, mean ± s.e.m. (*n* = 3 in each group); ***p < 0.0007; ****p < 0.0001; n.s. not significant, one-way ANOVA with Tukey’s test **f** In vitro kinase assay was performed using GST-PDCD5 as a substrate. Error bars, mean ± s.e.m. (*n* = 3 in each group); ****p < 0.0001. Statistical analysis was performed with one-way ANOVA with Tukey’s post hoc test (**d, f**). Radioactive in vitro kinase assay was performed in GST-PDCD5 or PDCD5^S119A^ with p38α recombinant protein. PDCD5 phosphorylation levels were analyzed by autoradiography (lower panel). Representative images and blots from three independent experiments are shown (**a**–**c**, **e**, **f**). Source data are provided in the Source Data file.

Casein kinase 2 (CK2) phosphorylates PDCD5 at Ser-119 upon genotoxic stress, which enhances its stability and promotes translocation into the nucleus^9,21^. Since PDCD5 expression was increased in the lungs of patients with IPF and in the lungs of mice with fibrosis, we examined whether CK2-mediated phosphorylation of PDCD5 at Ser-119 is required for PDCD5 stabilization and nuclear translocation in response to TGF-β_1_. First, we tested whether PDCD5 phosphorylation at Ser-119 is required for TGF-β-induced nuclear translocation of PDCD5. Wild-type PDCD5 (PDCD5^WT^) was rapidly translocated into the nucleus upon TGF-β_1_ treatment. However, a phospho-defective mutant (PDCD5^S119A^) did not translocate to the nucleus, even with TGF-β_1_ treatment. Importantly, a phospho-mimetic form of PDCD5 (PDCD5^S119E^) was localized primarily in the nucleus even without TGF-β_1_ treatment (Fig. 4a and Supplementary Fig. 11a). Together, these results suggest that phosphorylation of Ser-119 is required for nuclear translocation of PDCD5 in response to TGF-β.

Next, we examined whether CK2 phosphorylates PDCD5 in response to TGF-β. Unexpectedly, neither TBB, a specific inhibitor against CK2, nor knock down had an effect on PDCD5 expression in response to TGF-β (Fig. 4b and Supplementary Fig. 11b), indicating that another kinase is responsible for TGF-β-induced phosphorylation of PDCD5 at Ser-119. To identify the kinase responsible for PDCD5 phosphorylation at Ser-119, C22 cells were treated with kinase-specific inhibitors involved in signaling upstream of TGF-β. Among those inhibitors, the p38 inhibitor SB203580, significantly inhibited TGF-β-induced PDCD5 phosphorylation at Ser-119 (Fig. 4b, c and Supplementary Fig. 11c). TGF-β-induced *Postn* and *Ctgf* mRNA expression, as well as PDCD5 nuclear translocation were also blocked following treatment with SB203580 (Fig. 4d, e and Supplementary Fig. 11d). To examine whether p38 MAPK promotes the phosphorylation and stabilization of PDCD5, we assessed PDCD5 protein levels following the overexpression or knockdown of p38 MAPK in C22 cells. The stabilization of PDCD5 was regulated in a p38 MAPK expression-dependent manner (Supplementary Fig. 11e). Neither total nor phosphorylated levels of PDCD5 were increased upon TGF-β treatment in the RLE-6TN cell line. Moreover, p38 MAPK activation occurred after TGF-β treatment in C22 cells but not in the RLE-6TN line (Fig. 3a and Supplementary Fig. 9a). An in vitro kinase assay revealed that p38 directly phosphorylates PDCD5, and this phosphorylation was completely blocked by SB203580 treatment (Fig. 4f). These results suggest that p38 kinase directly phosphorylates PDCD5 at Ser-119 to promote its stability and nuclear translocation in response to TGF-β_1_ treatment.

### PDCD5-mediated matricellular proteins secretion in club cell promotes fibroblast activation

**Fig. 5:**
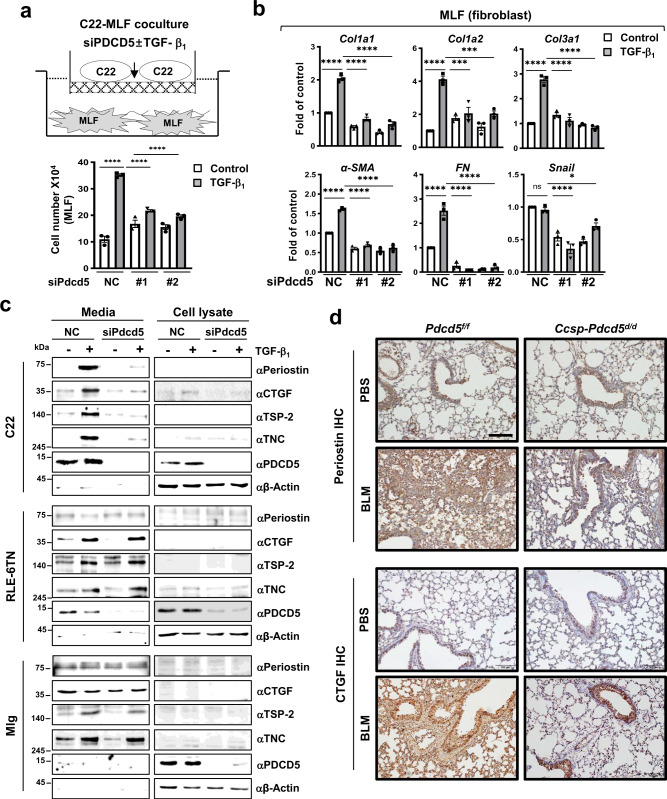
PDCD5 promotes fibroblast activation via secretion of matricellular proteins. **a** C22 cells were plated on transwells and transfected with Pdcd5 siRNAs. Mouse primary lung fibroblast cells (MLFs) were plated in 24-well plates a day before TGF-β_1_ treatment. Transwells were transferred to Mlg-containing 24-well plates. After 24 h TGF-β_1_ treatment, MLFs were counted using an automated cell counter (*n* = 3 in each group); ****p < 0.0001. **b** MLFs were cultured in C22 conditioned medium (CM). The levels of indicated genes were analyzed by qRT-PCR (*n* = 3 in each group) *p = 0.0265; ***p = 0.0002; ****p < 0.0001; n.s. not significant. Error bars represent mean ± s.e.m., statistical analysis was performed with one-way ANOVA followed by Tukey’s test (**a, b**). **c** C22, RLE-6TN, and Mlg cells were treated with TGF-β_1_ for 24 h. Enriched media and cell lysates were used for immunoblotting with indicated antibodies. Representative blots from three independent experiments are shown. **d** IHC with indicated antibodies was performed in the lung tissue from *Pdcd5*^*f/f*^ and *Ccsp-Pdcd5*^*d/d*^ mice. Scale bars = 100 μm. Representative images from three mice in each group are shown. Source data are provided in the Source Data file.

During pulmonary fibrosis, the abnormal airway epithelium secretes numerous paracrine factors that lead to deposition of pathological matrix by activated fibroblasts^22^. Therefore, we next examined the functional role of PDCD5 in epithelial club cells following fibroblast activation. Using epithelial-fibroblast co-culture analysis, we found that proliferation of primary mouse lung fibroblasts (MLFs) was significantly decreased following *Pdcd5* knockdown in epithelial C22 cells (Fig. 5a). Following Pdcd5 knockdown in C22 cells and co-culture with MLFs, we found that fibrotic target genes including *Collagen1a1 (Col1a1), Col1a2, Col1a3, alpha-Smooth Muscle Actin (α-SMA), Fibronectin (FN)*, and *Snail* were also reduced in MLF cells (Fig. 5b). We obtained similar results using Mlg fibroblasts co-cultured with C22 club cells (Supplementary Fig. 12a). However, Pdcd5 knockdown in AT2 cells had no effects on fibroblast proliferation or expression of fibrotic target genes (Supplementary Fig. 12b). Notably, PDCD5 knockdown in human MRC5 fibroblasts did not affect expression of fibrotic target genes, again verifying the selective role of PDCD5 in club cells (Supplementary Fig. 12c). These results were consistent with our in vivo data showing that Pdcd5 mediates lung fibrosis in a club cell-specific manner.

Given our data suggesting that TGF-β-induced PDCD5 expression modulates expression of matricellular genes, we next tested whether PDCD5 selectively regulates club cell-specific secretion of matricellular proteins in response to TGF-β. After 24 h of TGF-β_1_ treatment, media from C22, RLE-6TN, and Mlg cells were harvested and the level of matricellular proteins, including Periostin, CTGF, TSP-2, and TNC, was assessed by western blot. TGF-β dramatically increased secretion of matricellular proteins in C22, AT2, and fibroblast cells. However, Pdcd5 knockdown selectively reduced TGF-β-induced matricellular protein secretion in C22 club cells. In contrast, Pdcd5 knockdown in AT2 and Mlg fibroblast cell lines had no effect on secretion of matricellular proteins (Fig. 5c). Finally, we assessed changes in matricellular protein expression in the lung tissue of mice with BLM-induced fibrosis. Increased levels of BLM-induced CTGF, Periostin, and TNC were greatly decreased in the lung of *Ccsp-Pdcd5*^*d/d*^ mice when compared with those of control mice (Fig. 5d and Supplementary Fig. 13). Collectively, these data demonstrate that club cell-specific PDCD5 mediates fibroblast activation by promoting secretion of matricellular proteins, which eventually leads to pulmonary fibrosis.

## Discussion

Although the pathogenesis of IPF is not fully understood, it is believed that abnormal recovery after alveolar epithelium injury may eventually lead to development of the disease^23^. Secretion of various factors from damaged epithelial cells, including TGF-β, PDGF, and CTGF, promotes differentiation of fibrotic cells and accumulation of ECM^24^. Among lung epithelial cells, AT2 cells are the main bronchiolar epithelial cells responsible for activation of fibroblasts via a paracrine loop, and the majority of studies have therefore focused on this type cell^25^. On the other hand, club cells are non-ciliated bronchiolar exocrine cells that secrete a variety of proteins into the extracellular space to protect the bronchiolar epithelium from external damage^26^. A recent study showed that depletion of club cells using naphthalene reduced pulmonary fibrosis, demonstrating that club cells are important for development of lung fibrosis^27^. Club cells also play a progenitor role to maintain homeostasis of the bronchiolar walls^3^. Club cells are known to be widely located from trachea through bronchiole to bronchiolar alveolar duct junction. In particular, since these cells are located very close to the alveolar region, they migrate to the alveolar region in case of severe lung injury and have been reported to be involved in alveolar bronchiolization by inducing apoptosis in alveolar epithelial cells. Importantly, TRAIL-expressing club cells were detected within the affected alveolar epithelia in established fibrosis of IPF lungs^6^. However, the role of club cells in the pathogenesis of pulmonary fibrosis is still unclear. In this study, we found that PDCD5 mediates transcriptional activation of matricellular genes in club cells, which leads to activation of fibroblasts. We provided evidence that PDCD5 selectively mediates pulmonary fibrosis in a club cell-specific manner, but not in AT2 cells or fibroblasts. Consequently, we identified the physiological roles of PDCD5 in club cells and our results suggest that club cells have a significant functional role in pulmonary fibrosis.

Accumulating studies show that PDCD5 plays important roles in cell signaling by protein–protein interaction as a molecular chaperone. Our previous reports show that PDCD5 inhibits HDAC3–AKT interaction without affecting apoptosis of endothelial cells, which leads to inhibition of nitric oxide production in the vascular endothelium^10^. In contrast, in cancer cells, PDCD5 acts as a activator of apoptosis, disrupting HDAC3–p53 interaction to promote p53 activity^9^. In addition, PDCD5 interacts with TIP60, which enhances TIP60 stability, thereby promoting p53 signaling^28^. These observations suggest that PDCD5 is involved in regulation of various signaling pathways, including the cell death pathway. PDCD5 has six α-helices^8^; deletion of the first 30 N-terminal residues of PDCD5 abolishes interaction with p53, and introduction of the L6R PDCD5 mutation leads to dissociation from HDAC3, suggesting that the N-terminus of PDCD5 contains an α-helix that plays a key role in protein–protein interactions^9^. In this study, we found that PDCD5 directly interacts with β-catenin, but not Smad3, and mediates formation of the PDCD5/β-catenin/Smad3 complex. PDCD5 knockdown abrogated TGF-β-induced formation of the PDCD5/β-catenin/Smad3 complex and transcriptional activation of matricellular genes, indicating that PDCD5 acts as a molecular chaperone that mediates TGF-β signaling. Whether the α-helix-containing N-terminus of PDCD5 mediates the formation of the β-catenin/Smad3 complex is still unknown. Further study is required to understand the structural relationship of PDCD5 with the β-catenin/Smad3 complex.

Unlike previous findings, we found that p38 MAPK, but not CK2, directly phosphorylates PDCD5 at Ser-119 to promote its stability and nuclear translocation in response to TGF-β_1_ treatment; it is still unclear why CK2 failed to phosphorylate PDCD5 in the context of TGF-β signaling. Since the core enzymes of each cell act on the basis of respective signal transduction to regulate the function of target proteins, induction of PDCD5 phosphorylation at Ser-119 upon TGF-β cell signaling is more likely to be performed by p38 MAPK rather than CK2. It was recently reported that CK2 phosphorylates JAK2, a pro-fibrotic mediator in fibroblasts^29^; thus, CK2 may not be activated in club cells by TGF-β signaling or may mainly act as a mediator in fibroblasts. Additional studies will be needed to clarify this mechanism.

We found that PDCD5 promotes transcriptional activation of matricellular proteins. These secreted matricellular proteins interact with structural ECM proteins, such as collagen, which is required for formation of fibrils. Among those, CTGF is a key mediator of ECM production, leading to proliferation of lung fibroblasts and progression of lung fibrosis. CTGF does not behave like a traditional growth factor or cytokine because it does not have a specific receptor with a high affinity for signal transduction^30^; CTGF may be more accurately described as a matricellular protein that modulates cell–cell interactions to alter cellular phenotypes. Overexpression of CTGF promotes fibrosis in lung, kidney, and skin, and experimental reduction of CTGF suppresses fibrosis in animal models^31^. In the context of IPF therapy, a phase II clinical trial of Pamrevlumab (FG-3019), a therapeutic antibody against CTGF, has been completed, and Pamrevlumab is expected to be developed as a next-generation pulmonary fibrosis treatment due to its significant effects on lung fibrosis and low toxicity^32^. In our study, *Pdcd5* knockdown in club cells selectively diminished fibroblast proliferation and collagen synthesis. Furthermore, we observed that TGF-β-induced secretion of CTGF by club cells, but not AT2 cells, was greatly reduced following PDCD5 knockdown, indicating PDCD5 is a key regulator of CTGF secretion in club cells. Consistently, *Pdcd5* knockdown also blocked secretion of other matricellular proteins, including Periostin, CTGF, TSP-2, and TNC, indicating that PDCD5 is a master regulator of matricellular protein production in club cells. Recent reports suggest that various types of myofibroblasts are involved in induction of pulmonary fibrosis. This myofibroblast heterogeneity suggests that there is a limit to the development of lung fibrosis therapy by targeting a single protein^33^. Therefore, it is necessary to develop a treatment method that targets a core molecular circuit or pathway that can prevent activation of pathologic fibroblasts. Intriguingly, there are reports that serum PDCD5 levels are implicated in several diseases, such as endothelial dysfunction^10^, breast, gastrointestinal tract, lung cancer^34^, and chronic myeloid leukemia^35^. In accordance with these findings, we also observed that a significant amount of PDCD5 protein is present in C22 cell culture as well as cell lysates (Fig. 5c). Although clinical trials using CTGF antibodies are successfully ongoing, development of a diagnostic or targeting method of PDCD5 protein present in the blood may lead to advances in IPF therapy. In addition, α-SMA expression, which is a marker for EMT and myofibroblasts marker, is also up-regulated by PDCD5 in club cells. Intriguingly, we also observed that PDCD5 was highly co-localized with the fibroblast marker α-SMA in IPF patients, suggesting a plausible role for PDCD5 in myofibroblast activation during IPF development. Because club cells are known to act as stem cells, giving rise to ciliated cells during the regeneration of the bronchiolar epithelium^36^, further studies should examine whether PDCD5 mediates the EMT of club cells or activates myofibroblasts during the development of pulmonary fibrosis.

Collectively, our results demonstrate that PDCD5 is significantly elevated in the lungs of patients with IPF and in mice with pulmonary fibrosis. PDCD5 mediates TGF-β-induced matricellular protein secretion via formation of PDCD5/β-catenin/Smad3 complex in club cells, which eventually leads to activation of fibroblasts and ECM accumulation. Our results suggest that PDCD5 acts as a pulmonary fibrosis mediator in a club cell-specific manner. Therefore, our study provides a conceptual framework for understanding the interconnectivity between club cells and pulmonary fibrosis, with implications for the diagnosis and treatment of IPF.

## Methods

### Patient samples

Human lung tissue samples were obtained from the tissue bank at Severance Hospital (Seoul, Korea). A total of 19 samples from IPF patients between September 2015 and December 2016 were included in the study. The patients fulfilled the criteria of the American Thoracic Society and European Respiratory Society^1^, and diagnosis of IPF was supported by history, physical examination, pulmonary function studies, chest high-resolution computed tomography, and corroborated by video-assisted thoracoscopic lung biopsy or transplant explants. Disease staging was performed using gender–age–physiology (GAP) index and categorized into stages I to III^37^. A total of 10 control samples, including lung samples with normal histology from patients with lung cancer, were also obtained from the same tissue bank. Informed consent was obtained from all patients and controls. This study was approved by the ethical committee of the institutional review board of Severance Hospital and in accordance with regulations (protocol no. 4-2016-0453).

### Animal studies

All animal experiments were conducted in accordance with standard operating guidelines with the approval of Yonsei University College of Medicine Institutional Animal Care and Use Committee (IACUC No. 2015-0047). Six- to 8-week-old C57BL/6 male mice were used for this study (Orient Bio. Korea). We crossbred *Pdcd5*^*f/f*^ mice^9^ with *Scgb1a1-CreER*^*TM*^ mice^14^ (The Jackson Laboratory, Sacramento, CA, USA) or *Sftpc-CreER*^*T2*17^ (gifted from Brigid L.M. Hogan, Duke University Medical Center) to generate *Pdcd5*^*f/f*^*;Scgb1a1*^*Cre/+*^ mice (*Ccsp-Pdcd5*^*d/d*^) or *Pdcd5*^*f/f*^*;Sftpc*^*Cre/+*^ (*Spc-Pdcd5*^*d/d*^) mice. *ROSA26-mTmG* mouse^18^ was crossed to *Ccsp-Pdcd5*^*d/d*^ or *Spc-Pdcd5*^*d/d*^ mice to visualize *Cre*-mediated excision of *Pdcd5* in club cells or AT2 cells. To induce knockout of *Pdcd5*, 10 mg/kg hydroxytamoxifen (4-OHT; Sigma-Aldrich, St. Louis, MO, USA) was injected into 8-week-old male mice three or four times every other day. Three days after the last injection, mice were injected with saline (vehicle control) or 4 mg/kg BLM (Santa Cruz Biotechnology, Santa Cruz, CA, USA) intratracheally. Mice were sacrificed after receiving bleomycin and BAL fluid and lung tissues were harvested. Mice were housed in a specific pathogen-free animal facility, with controlled temperature (~24 °C) and humidity under 12 h light/dark cycle (lights on at 8 a.m.) and had free access to food and water.

### TGF-β_1_ transgenic mice

Eight-week-old TGF-β_1_ transgenic mice (*Ccsp-rtTA-tTS-TGF-β*_*1*_) and littermate controls were randomized to normal water or water containing 0.5 mg/ml of doxycycline (Dox) (Sigma-Aldrich) to induce TGF-β_1_ overexpression, as described previously^38^. To generate club cells with *Pdcd5* knockout and TGF-β_1_ overexpression, *Ccsp-rtTA-tTS-TGF-β*_*1*_ *(Ccsp-TGFβ*_*1*_-TG) mice were bred to *Pdcd5*^*f/f*^ or *Ccsp-Pdcd5*^*d/d*^ mice. After four total injections of 10 mg/kg 4-OHT to *Ccsp-TGFβ*_*1*_-TG with *Ccsp-Pdcd5*^*d/d*^ mice, administered every other day, and then Dox was administered for 4 weeks (Supplementary Fig. 5b).

### Mouse primary cell sorting

Eight-week-old mice were injected with saline or 4 mg/kg BLM, intratracheally. Mice were sacrificed 7 days after BLM injection, and saline perfused lungs were treated using a mouse lung dissociation kit (Miltenyi Biotec, Auburn, CA, USA), in the gentleMACS^TM^ Dissociator (Miltenyi Biotec). Red blood cells (RBCs) were lysed with RBC lysis solution (Miltenyi Biotec), and isolated cells from the mouse lung were double stained with anti-CCSP/anti-EpCAM for club cells or anti-CD74/anti-EpCAM for AT2 cells. Isotype control antibodies (IgG2a κ-FITC and IgG2a κ-APC; eBioscience, San Diego, CA) were used to establish gating parameters for positively stained cells. Double-positive populations were sorted using a BD FACSAria^TM^ III cell sorter (BD Biosciences, San Jose, CA, USA). Sorted double-positive cells were subjected to an RNeasy Micro Kit (Qiagen, Germany) to obtain mRNA for qRT-PCR assays or plated on chamber slides (SPL Life Sciences, Korea) for in situ proximity ligation assay (PLA) analysis.

### Soluble collagen assay

Collagen content in the mouse lung was determined biochemically using the Sircol Collagen Assay kit (Biocolor, Carrickfergus, UK) according to the manufacturer’s instructions.

### Immunohistochemistry

Mouse lung tissues from each group were fixed in formaldehyde (10% w/v) for 48 h at room temperature and embedded in paraffin. Tissue sections were processed for MTS and IHC. Immunohistochemical staining was performed as previously described^10^. The following antibodies were used: anti-PDCD5 (Proteintech, Rosemont, IL, USA), anti-CTGF, anti-Periostin, and anti-TNC were purchased from Abcam (Cambridge, MA, USA). Normal rabbit IgG (Santacruz) was used for non-immune control. To visualize bound antibodies, an Immunostaining-DAKO Envision Plus kit (DAKO, Santa Cruz, CA, USA) was used.

### Cell culture and reagents

The immortalized mouse club cell line, C22 (Sigma-Aldrich) was maintained as previously described^39^. Briefly, cells were maintained in permissive conditions (Dulbecco’s modified Eagle medium (Corning, Manassas, VA, USA) supplemented with 2% fetal bovine serum, penicillin [100 U/ml], streptomycin [100 μg/ml], amphotericin B [250 μg/ml], endothelin-1 [0.25 μg/ml], interferon-γ [100 U/ml], insulin [10 μg/ml], transferrin [5 μg/ml], endothelial cell growth supplement [7.5 μg/ml], epidermal growth factor [0.025 μg/ml], hydrocortisone [0.36 μg/ml], and T3 [0.02 μg/ml]) at 33 °C prior to experiments, and then transferred to non-permissive conditions (without interferon-γ) at 37 °C to inactivate large T-antigen. The rat RLE-6TN AT2 cell line (kindly presented from Sang Myun Park Ajou University, Korea), mouse Mlg fibroblast cell line, and human MRC5 fibroblast cell line (Korean cell line bank, Seoul, Korea) were maintained in Dulbecco’s modified Eagle’s medium supplemented with 10% fetal bovine serum and 1% antibiotic/antimycotic solution (Corning) at 37 °C in 5% CO_2_. Primary mouse fibroblast cells were obtained from Cell Biologics (Chicago, IL, USA). TGF-β_1_ was purchased from Prospec (East Brunswick, NJ, USA). SB203580 (p38 inhibitor) and SP600125 (JNK inhibitor) were purchased from Selleckchem (Houston, TX, USA). TBB (4,5,6,7-tetrabromobenzotriazole) were purchased from Sigma-Aldrich.

### Fluorescence imaging

Tissue slides were rehydrated in xylene and ethanol and citrate buffer (DAKO) was used for antigen retrieval. Anti-PDCD5 (R&D Systems, MN, USA), anti-p-PDCD5 (ref. ^9^), anti-Flag (Sigma-Aldrich), anti-CCSP (Millipore), anti-pro-SP-C (Santacruz), and anti-PDPN, anti-Scgb3a2 (R&D Systems), anti-Muc5AC, anti-Foxj1 (Thermo Fisher Scientific), anti-α-SMA, and anti-Ki67 (Abcam) antibodies were incubated at 4 °C overnight and stained with Alexa Fluor 488-, Alexa Fluor 549-, or Alexa Fluor-647-conjugated goat anti-rabbit or anti-mouse secondary antibodies (Thermo Fisher Scientific). Nuclei were stained with DAPI (Abcam). Slides were mounted using fluorescence mounting medium (DAKO). Cryosections from unfixed lungs were embedded in OCT compound. Frozen sections (5-μm-thick) were cut with a cryostat at −20 °C and mounted with DAPI-containing mounting medium (Sigma-Aldrich). TUNEL-positive cells were detected using In Situ Cell Death Detection Kit (Sigma-Aldrich).

Cells were cultured on chamber slides (SPL Life Sciences, Korea) and fixed in 4% paraformaldehyde for 30 min at room temperature. After washing with phosphate-buffered saline (PBS), fixed cells were permeabilized with 0.1% Triton X-100) and incubated for 20 min with 3% bovine serum albumin to block nonspecific antibodies. Cells were incubated with primary antibodies for overnight, and then incubated with secondary antibodies. The slides were mounted with DAPI-containing mounting medium (Abcam), then imaged using an LSM 710 Laser Scanning Microscope (Carl Zeiss, Germany) and co-localization and mean intensity profiles were analyzed using ZEN 3.0 (black edition) software.

### Western blot analysis and immunoprecipitation

Cells were lysed in lysis buffer (20 mM Tris-Cl, 150 mM NaCl, 1% Triton X-100, 1.5% MgCl_2_, 1 mM ethylenediaminetetraacetate, 1 mM Na_2_VO_4_, 1 mM phenylmethylsulfonyl fluoride, and protease inhibitor cocktail at pH 7.5). Lysates were briefly vortexed and cleared by centrifugation at 16,000 × *g* for 20 min at 4 °C; supernatants were collected and transferred to fresh tubes. Protein concentrations were determined using a 660-nm protein assay reagent (Thermo Fisher Scientific, Rockford, CT, USA). The samples were then precleared with protein A/G agarose beads (Santa Cruz) for 2 h and precipitated using protein A/G agarose beads. Equal amounts of protein extracts and immunoprecipitation products were subjected to electrophoresis on sodium dodecyl sulfate-polyacrylamide gels and transferred to nitrocellulose transfer membranes (Whatman, Dassel, Germany). The membranes were blocked in Tris-buffer (pH 7.4) containing 0.1% (v/v) Tween 20 (Sigma-Aldrich) and 5% (w/v) nonfat Difco skim milk (BD Biosciences, San Jose, CA, USA), and then probed with primary antibodies. The following antibodies were used: anti-p-PDCD5 Ser-119 (ref. ^9^), anti-PDCD5 (Proteintech; R&D Systems, MN, USA; Abcam); Anti-Smad2, anti-Smad3, anti-p-Smad2, anti-p-Smad3, anti-p38, anti-pp38, and Lamin A/C antibodies (Cell Signaling, Danvers, MA, USA); anti-Periostin, anti-CTGF, anti-TSP-2, and anti-TNC (Abcam); anti-HA and anti-GAPDH (Santa Cruz), anti-β-catenin (BD Biosciences), anti-FLAG and anti-β-actin (Sigma-Aldrich). The membranes were then washed with 1× PBST, incubated with the appropriate secondary anti-rabbit or anti-mouse horseradish peroxidase-conjugated antibodies (Thermo Scientific, Rockford, IL, USA) for 1 h, and visualized using the LAS-3000 system (Fujifilm, Stamford, CT, USA) with an enhanced chemiluminescence detection reagent (Thermo Scientific).

### RNA isolation and quantitative RT-PCR

Total RNA from cells was prepared using Ribospin II (GeneAll Biotech, Korea), and cDNA was synthesized using Cellscript (CellSafe, Korea). For RNA sequencing, quality was assessed using an Agilent 2100 bioanalyzer and RNA 6000 Nano Chip (Agilent Technologies, Amstelveen, The Netherlands). PCR primer sequences for qRT-PCR are listed in Supplementary Table 2. We normalized the concentration of cDNA using mouse *Tuba-1b*, rat *18s, or* human *ACTB*. qRT-PCR analyses were performed using SYBR Green PCR master mix reagents and an ABI Prism 7700 sequence detection system (Applied Biosystems, Carlsbad, CA, USA). All reactions were carried out in triplicate. Relative expression levels and standard deviation were calculated using the comparative method.

### Library preparation and sequencing

For control and test RNAs, library construction was performed using a QuantSeq 3′ mRNA-seq Library Prep kit (Lexogen, Inc., Austria) according to the manufacturer’s instructions. In brief, 500 ng of each RNA was prepared and an oligo-dT primer containing an Illumina-compatible sequence at its 5′ end was hybridized to the RNA and reverse transcription was performed. After degradation of the RNA template, second strand synthesis was initiated using a random primer containing an Illumina-compatible linker sequence at its 5′ end. The double-stranded library was purified using magnetic beads to remove all reaction components. The library was amplified to add the complete adapter sequences required for cluster generation. The finished library therefore consisted of purified PCR components. High-throughput sequencing was performed as single-end 75 sequencing using NextSeq 500 (Illumina, Inc., USA).

### Data analysis

QuantSeq 3′ mRNA-seq reads were aligned using Bowtie2 (version 2.3.4.3)^40^. Bowtie2 indices were either generated from the genome assembly sequence or the representative transcript sequences for aligning to the genome and transcriptome. The alignment file was used for assembling transcripts, estimating abundances, and detecting differential gene expression. Differentially expressed genes were determined based on counts from unique and multiple alignments using coverage in Bedtools (version 2.26)^41^. The RC (read count) data were processed using the quantile normalization method with EdgeR within R version 4.0.3 (R development Core Team, 2016) using Bioconductor^42^. GSEA was performed using GSEA version 4.0.1 software (Broad Institute). Gene sets were obtained from the Molecular Signatures Database (MSigDB, version 7.0). Gene classification was based on searches done by DAVID (vesion 6.8; http://david.abcc.ncifcrg.gov/).

### ChIP assay

ChIP was carried out on 10^7^ C22 cells using a Pierce Agarose ChIP kit (Thermo Fisher Scientific) according to the manufacturer’s instructions. The nuclear lysates were precipitated with anti-PDCD5 (R&D Systems), anti-p-Smad3 (Cell Signaling), anti-β-catenin (BD Biosciences), or normal rabbit IgG (Thermo Fisher Scientific) antibodies overnight at 4 °C together with ChIP-grade protein A/G agarose beads. The beads were then washed, eluted, and treated with proteinase K. The recovered DNA was amplified by qRT-PCR using primers listed in Supplementary Table 2.

### In situ PLA analysis

In situ PLA analysis (Sigma-Aldrich) was carried out following the manufacturer’s instructions. Cells were fixed with 4% paraformaldehyde, washed with PBS, and blocked with blocking solution. After the application of p-Smad3 and β-catenin antibodies, cells were incubated with PLUS and MINUS secondary PLA probes based on species and then subjected to ligation and amplification using the provided reagents. The samples were mounted with Duolink mounting medium and analyzed using an LSM 710 Laser Scanning Microscope (Carl Zeiss).

### p38 in vitro kinase assay

The p38 in vitro kinase assay was performed using the p38α kinase assay kit (Promega, Madison, WI, USA) according to the manufacturer’s instructions. Purified GST, GST-PDCD5, and GST-PDCD5 S119A were used as substrates. For radioactive in vitro kinase assay, GST-fusion proteins were incubated with 1 μg of recombinant p38α (Promega) in the presence of kinase reaction buffer (12.5 mM MgCl_2_/100 mM ATP plus 100 μCi of [γ-^32^P]-ATP) in a total volume of 50 μl for 2 h at 30 °C. Reaction were terminated by washing twice with 1× kinase buffer. Samples were resuspended in 15 μl 5× SDS sample loading buffer and boiled for 5 min. After electrophoresis, SDS polyacrylamide gel was stained with Coomassie blue and dried, and the phosphorylated products were visualized by autoradiography.

### Media concentration and co-culture

Medium from C22, RLE-6TN, or Mlg cells cultured in 60-mm cell culture dishes was harvested, passed through a 0.45 μm filter (Sartorious, Stonehouse, UK), and used as conditioned medium. Additionally, culture medium was applied to a VIVASPIN6 column (Sartorious) and centrifuged for 3 h at 10,000 × *g*; concentrated protein was calculated using a protein assay, and then immunoblotted using antibodies. For co-culture, C22 or RLE-6TN cells were plated on Transwell inserts (Corning) with 0.45 μm pores, which allowed free flux of secreted proteins between the two cell types.

### Statistical analysis

Statistical analyses of human data were conducted with R (version 3.3.2; R Foundation for Statistical Computing, Vienna, Austria). Continuous data were reported as the mean (± standard deviation [SD]) or median (interquartile range). Comparisons of two groups were performed using a Student’s *t*-test or Mann–Whitney *U*-test for continuous variables, and the chi-square test or Fisher’s exact test was used to analyze categorical variables. Experimental results were visualized and analyzed with Prism version 9 (GraphPad Software) and are depicted as the mean ± standard error of the mean (s.e.m.). For a two-group comparison, a Mann–Whitney *U*-test or Student’s *t*-tests were used. *P* values < 0.05 were considered statistically significant. When more than two groups of samples were compared, a one-way ANOVA was used. Tukey’s range test was used for post hoc analyses of ANOVA.

### Reporting summary

Further information on experimental design is available in the Nature Research Reporting Summary linked to this paper.

## Supplementary information

Supplementary Information

Reporting Summary

## Data Availability

RNA-Sequencing data have been deposited in the NCBI Gene Expression Omnibus and are accessible through GEO Series, using accession number GSE143841. All other data supporting the findings from this study are available on reasonable request. Source data are provided with this paper.

## Source data

Source Data
