# Supplementary material for: Club cell-specific role of programmed cell death 5 in pulmonary fibrosis
Source: Nat Commun. 2021 May 19;12:2923. doi: 10.1038/s41467-021-23277-8 (PMC8134485; doi:10.1038/s41467-021-23277-8)
Supplement: Supplementary file 1 — Supplementary Information [file 41467_2021_23277_MOESM1_ESM.pdf]

Supplementary Information for

Club cell-specific role of programmed cell death 5 in pulmonary fibrosis

Soo-Yeon Park[1,†], Jung Yeon Hong[2,†], Soo Yeon Lee[1], Seung-Hyun Lee[1], Mi Jeong Kim[1], Soo Yeon Kim[2], Kyung Won Kim[2], Hyo Sup Shim[3], Moo Suk Park[4], Chun Geun Lee[5,6], Jack A. Elias[5], Myung Hyun Sohn[2,*] and Ho-Geun Yoon[1,*]

*Correspondence to: YHGEUN@yuhs.ac, mhsohn@yuhs.ac

Contents:

Supplementary Table 1, 2
Supplementary Figures 1 to 14

Supplementary Table 1. Demographic and clinical data of study population

	Control (n = 10)	IPF (n = 19)	*P*-value
Age, years	63.0 (61.0-76.0)	62.0 (55.0-65.0)	0.333
Sex, M (%M)	3 (30.0)	15 (78.9)	0.029
Height, cm	160.7 ± 7.6	165.4 ± 5.3	0.067
Weight, kg	65.8 ± 14.6	63.4 ± 10.9	0.806
IPF stage, I:II:III (n)	N/A	0:9:10	
FVC, L	2.8 ± 0.6	1.4 ± 0.6	< 0.001
FVC, % predicted	91.8 ± 8.8	37.0 ± 12.3	< 0.001
FEV_1, L	2.1 ± 0.6	1.2 ± 0.5	< 0.001
FEV_1, % predicted	97.2 ± 12.3	44.4 ± 15.1	< 0.001
DLCO, mL/mmHg/min	17.1 ± 2.9	5.3 ± 2.5	< 0.001
DLCO, % predicted	92.7 ± 11.6	26.6 ± 9.1	< 0.001
PDCD5 intensity/H&E	0.6 (0.4-0.9)	1.7 (1.6-2.4)	< 0.001

FVC, Forced Vital Capacity; FEV_1, Forced Expiratory Volume in 1 s; DLCO, Diffusing Capacity for Carbon Monoxide

N/A, not applicable.

Data are given as number (%), mean (± standard deviation), or median (interquartile range), as appropriate.

Supplementary Table 2. Sequences of RT-PCR primers, genotyping primers, siRNAs, ChIP assay primers

Target	Sense (5' ⇒ 3')	Antisense (5' ⇒ 3')
**qRT-PCR primer**		
**mouse Postn**	GCAAACCACTTTCACCGACC	GAGGTCGCTAAGGCCAACTT
**mouse Ctgf**	CCACCCGAGTTACCAATGAC	GTGCAGCCAGAAAGCTCA
**mouse Thbs2**	GACCAAACCTACGCTGGTGGA	AGCACATTGCTGGAGCTGGA
**mouse Tnc**	TGCAACGACTTCCTTTGCAC	GGCTCGGAGAATGACCATGT
**mouse α-SMA**	GTGACTCACAACGTGCCTATC	CTCGGCAGTAGTCACGAAGG
**mouse Serpine1**	GACACCCTCAGCATGTTCATC	AGGGTTGCACTAAACATGTCAG
**mouse col1a1**	ATGGATTCCCGTTCGAGTACG	TCAGCTGGATAGCGACATCG
**mouse col1a2**	TGCAGTAACTTCGTGCCTAGC	ACGTGGTCCTCTGTCTCCA
**mouse col3a1**	CTAAAATTCTGCCACCCCGAA	AGGATCAACCCAGTATTCTCCACTC
**mouse FN**	AAGACCATACCTGCCGAATG	GAACATGACCGATTTGGACC
**mouse Snail**	TCTGAAGATGCACATCCGAAGCCA	AGGAGAATGGCTTCTCACCAGTGT
**mouse Pdcd5**	GCAAAGGGAAGCAGAAATGA	CCTTCCCACTTAGCTGTCCA
**mouse Scgb1a1**	CATCATGAAGCTCACGGAGAAAATC	AAAGAGGAAGGAGGGGTTGGT
**mouse Sftpc**	AGCATCCACAGGGTCGGTAG	GCCCGTAGGAGAGACACCTT
**mouse Tuba-1b**	GTGCATCTCCATCCATGTTG	GTGGGTTCCAGGTCTACGAA
**rat Postn**	TGGTGAGAGGAAGCAAGCAG	GCTGAAGCGCTTATCTTGGC
**rat Ctgf**	ACATTAAGAAGGGCAAAAAGTGCAT	CACCCCACAGAACTTAGCCC
**rat Thbs2**	CAAGGACATGCAGTTTGGGC	GAAGACCAGGGTGACCAGTG
**rat Tnc**	AGGGGTAGACTGCTCTGAGG	TCCCGGATGGTGGTAGATGT
**rat Serpine1**	CGTCTTCCTCCACAGCCATT	GTTGGATTGTGCCGAACCAC
**rat α-SMA**	TTCATTGGAATGGAGTCGGCG	CTGTCAGCAATGCCTGGGTA
**rat Pdcd5**	ATGGCGGACGAAGAACTTGAG	GGGCTGACTGATCCAGAACT
**Rat 18s**	CATTCGAACGTCTGCCCTAT	GTTTCTCAGGCTCCCTCTCC
**human COL1a1**	CCTCAAGGGCTCCAAC	GGTTTTGTATTCAATCACTGTCTTGC
**human COL1a2**	TGCAGTAACTTCGTGCCTAGC	ACGTGGTCCTCTGTCTCCA
**human COL3a1**	TGGTCTGCAAGGAATGCCTGGA	TCTTTCCCTGGGACACCATCAG
**human α-SMA**	CTGGCATCGTGCTGGACTCT	GATCTCGGCCAGCCAGATC
**human FN**	GAGCTATTCCCTGCACCTGATG	CGTGCAAGGCAACCACACT
**human Snail**	CCCCAATCGGAAGCCTAACT	GGACAGAGTCCCAGATGAGC
**human Pdcd5**	GATCCTGGTGATGCAGCCC	CTTCCCACTTAGCTG
**human ACTB**	TGCGTGACATTAAGGAGAAG	GTCAGGCAGCTCGTAGCTCT
**Genotyping PCR primer sequence**		
**PDCD5 1st LoxP**	CTTGGGACAAACGCTAGTGG	GGAAACTGGACCTCACCAAA
**PDCD5 2nd LoxP**	GTGGGCTCTATGGCTTCTGA	GCAGGGGTAAAACAGCAGAG
**mutnat sense**	CAGTAAATACCCACGGAGTT	
**Scgb1a1-Cre**	ACTCACTATTGGGGGTGTGG	AGGCTCCTGGCTGGAATAGT
**mutant antisense**		CCAAAAGACGGCAATATGGT
**Sftpc-Cre**	TGGTTCCGAGTCCGATTCTTC	CCTTTTGCTCTGTTCCCCATTA
**mutant sense**	TCGCCTTCTATCGCCTTCTTG	
**TGF-β1**	CAGCTGTACATTGACTTCC	CACGTAGTACACGATGGGCA
**rtTA**	GTCGCTAAAGAAGAAAGGGAAACAC	TTCCAAGGGCATCGGTAAACATCTG
**mT/mG**	CTCTGCTGCCTCCTGGCTTCT	CGAGGCGGATCACAAGCAATA
**mutant antisense**		TCAATGGGCGGGGGTCG
**siRNA sequence**		
**Negative Control**	CCUCGUGCCGUUCCAUCAGGUAGUU	CUACCUGAUGGAACGGCACGAGGUU
**siPDCD5#1**	GUAACUUAGCACUUGUAAAUU	UUUACAAGUGCUAAGUUACUU
**siPDCD5#2**	GCAGAAAUGAGAAACAGUAUU	UACUGUUUCUCAUUUCUGCUU
**siPDCD5#4**	CAACAACAGUGAAAUUCAAUU	UUGAAUUUCACUGUUGUUGUU
**siCK2α (mouse)**	CUAUCAGAUGUAUGAUUAUUU	AUAAUCAUACAUCUGAUAGUU
**sip38 (mouse)**	GACUGUGAGCUCAAGAUUC	GAAUCUUGAGCUCACAGUC
**ChIP assay primer sequence**		
**mouse Ctgf promoter**	ATCAGGAGGGGTGAGAAGATGA	GATCGGGACGCGGTAGGA
**mouse Postn promoter**	CACAAGTGCCCTGGAAGGAA	ACTGTGTTGTTTGGCTGTGT

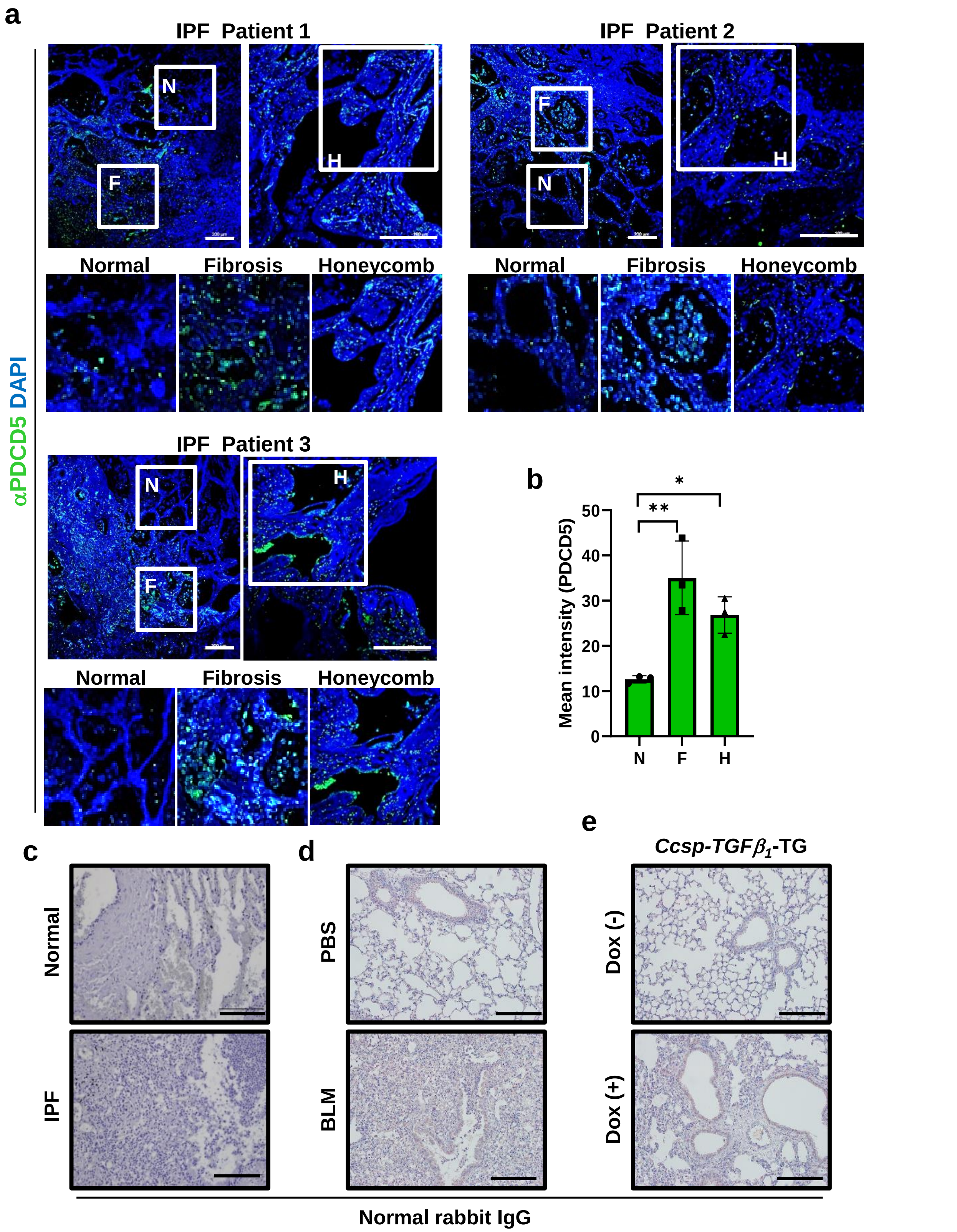

Supplementary Fig. 1. Increased expression of PDCD5 in the honeycomb region of IPF patients. a,b Immunofluorescence staining against PDCD5 (green) was performed in IPF patients. Increased PDCD5 intensity was detected in fibrotic area (F) and honeycomb cysts (H) compare with the normal (N) region. Scale bars = 200 μm. Error bars represent mean ± s.e.m. (n = 3/group); *p = 0.0372; **p = 0.0048, one-way ANOVA followed by Tukey's test. **c–e** IHC was performed using normal rabbit IgG in IPF patients **(c)**, BLM-induced wild type mouse lung **(d),** and Dox-induced TGF-β transgenic mouse lung **(e)**. Representative images from three independent experiments are shown. Scale bars = 200 μm. Source data are provided in the Source Data file.

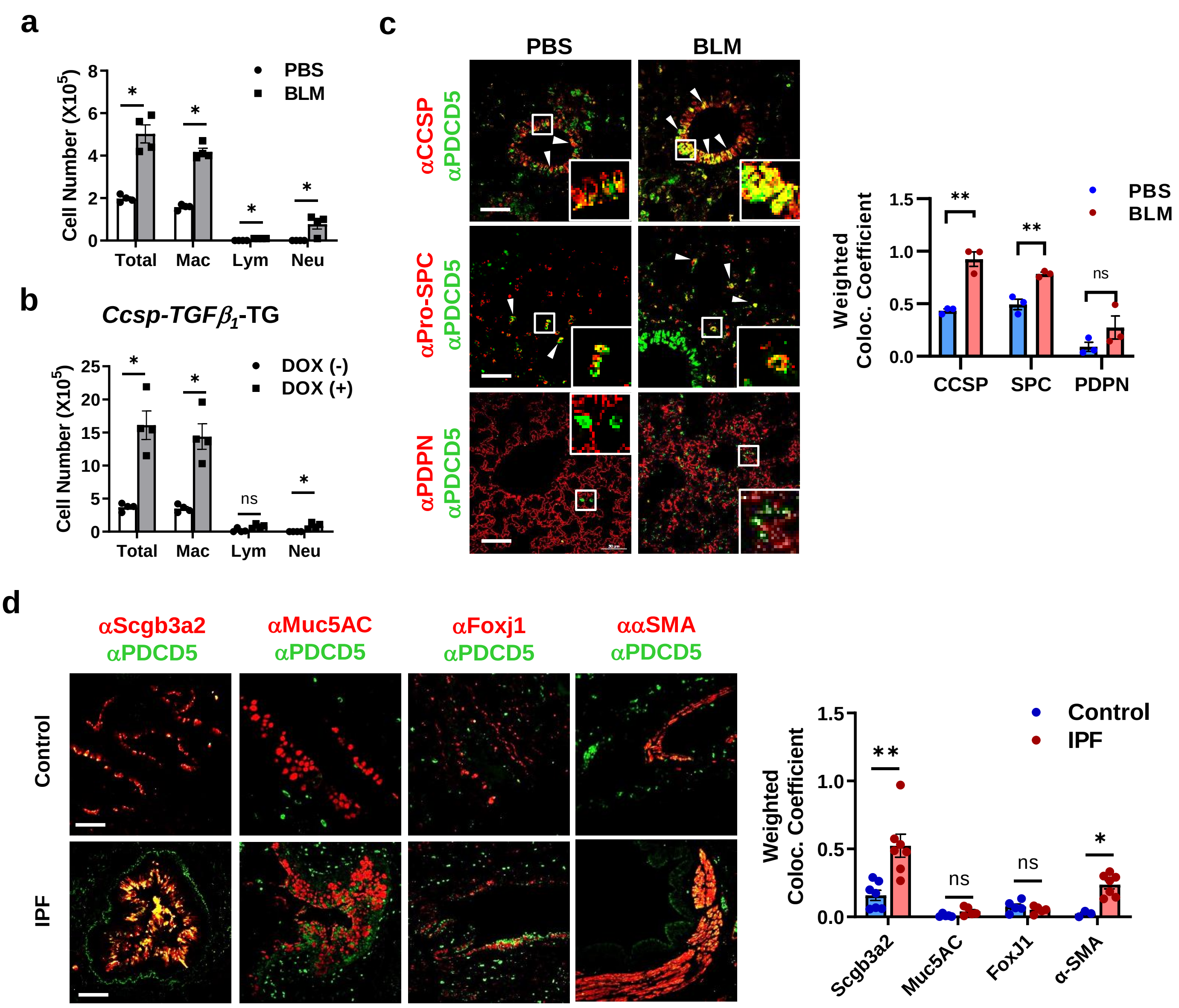

Supplementary Fig. 2. Expression patterns of PDCD5 in the lung from lung fibrosis mice models and IPF patients. a,b Total cells, Mac (macrophages), Lym (lymphocytes), and Neu (neutrophils) enumerated in the lungs from BLM-induced wild type mice **(a)** and Dox-induced TGFβ-TG mice **(b)**. Error bars represent mean ± s.e.m. (n = 4 mice/group), *$p < 0.0294$; ns, not significant, two-tailed Mann-Whitney test. **c** In wild type mice, Co-IF staining was performed in BLM-induced lung tissues using the indicated antibodies. White arrowheads indicate co-localization regions (Scale bars = 50 μm). Error bars represent mean ± s.e.m. (n = 3/group). **$p < 0.0056$; ns, not significant, unpaired two-tailed t test. **d** Double staining against PDCD5 and each indicated cell marker in IPF patients. Representative immunofluorescence images of lung sections of IPF patients co-stained for PDCD5 and makers of club cells (Scgb3a2, n = 7/group), goblet cells (Muc5AC, n = 5/group), ciliated cells (Foxj1, n = 5/group) and fibroblast cells (αSMA, n = 5 in control; n = 7 in IPF). Weighted co-localized coefficients were calculated using ZEN software. Scale bars = 50 μm. Error bars represent mean ± s.e.m. *$p = 0.0167$; **$p = 0.0012$; ns, not significant, two-tailed Mann-Whitney test. Source data are provided in the Source Data file.

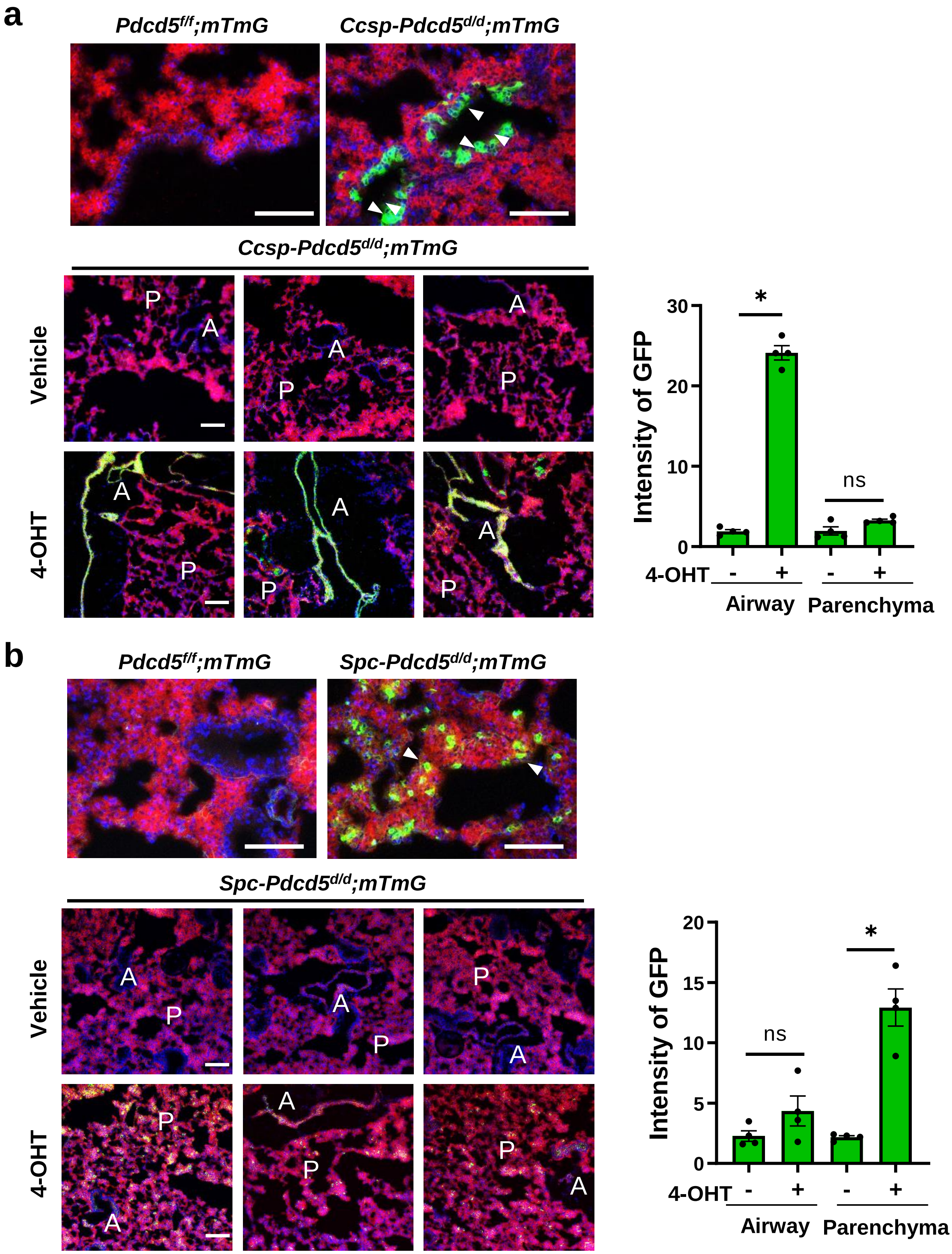

Supplementary Fig. 3. Assessment of 4-OHT-induced *Cre* activity in *Ccsp-Pdcd5$^{d/d}$;mTmG and Spc-Pdcd5$^{d/d}$;mTmG* mice. a, b Green fluorescent signals (white arrowheads) in mTmG images from *Ccsp-Pdcd5$^{d/d}$;mTmG* mice (**a**) and *Spc-Pdcd5$^{d/d}$;mTmG* mice (**b**) indicated positive *Cre* activity. 4-OHT was injected into each mouse a total of 3 times, every other day. Three days after the last injection, lung tissue was harvested. Each frozen block was fixed with acetone solution and mounted with DAPI-containing mounting solution. mTmG images were detected using a confocal microscope. The GFP intensity values of the Airway (A) and Parenchyma (P) were calculated with ZEN 3.0 software. Scale bars = 100 µm. Error bars represent mean ± s.e.m. (n = 4/group); *p = 0.0286 ; ns, not significant, two-tailed Mann-Whitney test. Source data are provided in the Source Data file.

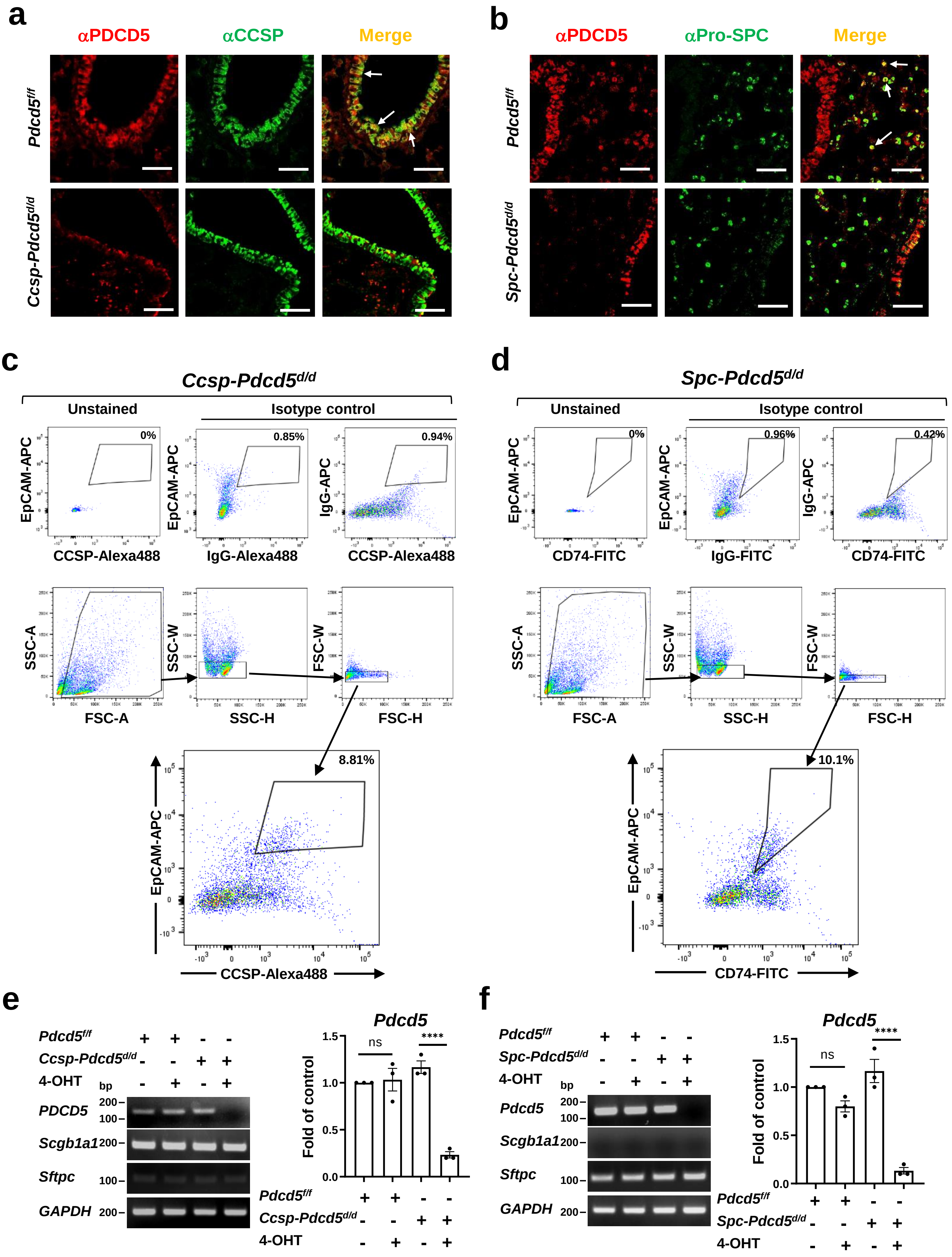

Supplementary Fig. 4. See next page for legend

Supplementary Fig. 4. Validation of *Pdcd5* ablation in the lungs of *Ccsp-Pdcd5$^{d/d}$* and *Spc-Pdcd5$^{d/d}$* mice. a, b Co-immunofluorescence analysis of PDCD5 and cell-specific markers was performed in the lungs of *Ccsp-Pdcd5$^{d/d}$* (CCSP) mice (**a**) and *Spc-Pdcd5$^{d/d}$* (Pro-SPC) mice (**b**) to verify deletion of Pdcd5. White arrows indicate co-localization regions. Scale bars = 50 μm. Representative images for three mice from each group are shown. **c, d** Gating and sorting strategies for isolation of primary club cells (CCSP^{+}EpCAM^{+}) or AT2 cells (CD74^{+}EpCAM^{+}) were shown. Isotype controls were used in all gates to control for unspecific background staining. Cells were selected on the basis of FSC and SSC (gated cell population). Aggregates and doublets are excluded using side and forward scatter areas versus their respective width parameters. Next, gating on club cell (CCSP^{+}EpCAM^{+}) or AT2 cells (CD74^{+}EpCAM^{+}) was used for sorting of double-positive population. **e, f** Isolated club cells from *Pdcd5$^{f/f}$* or *Ccsp-Pdcd5$^{d/d}$* mice **(c),** and AT2 cells from *Pdcd5$^{f/f}$* or *Spc-Pdcd5$^{d/d}$* mice **(d)** after vehicle or 4-OHT treatment were used to perform RT-PCR and quantitative RT-PCR analysis. Error bars represent mean ± s.e.m. (n = 3 mice in each group); **p* < 0.0001; ns, not significant, one-way ANOVA with Tukey's test. Source data are provided in the Source Data file.

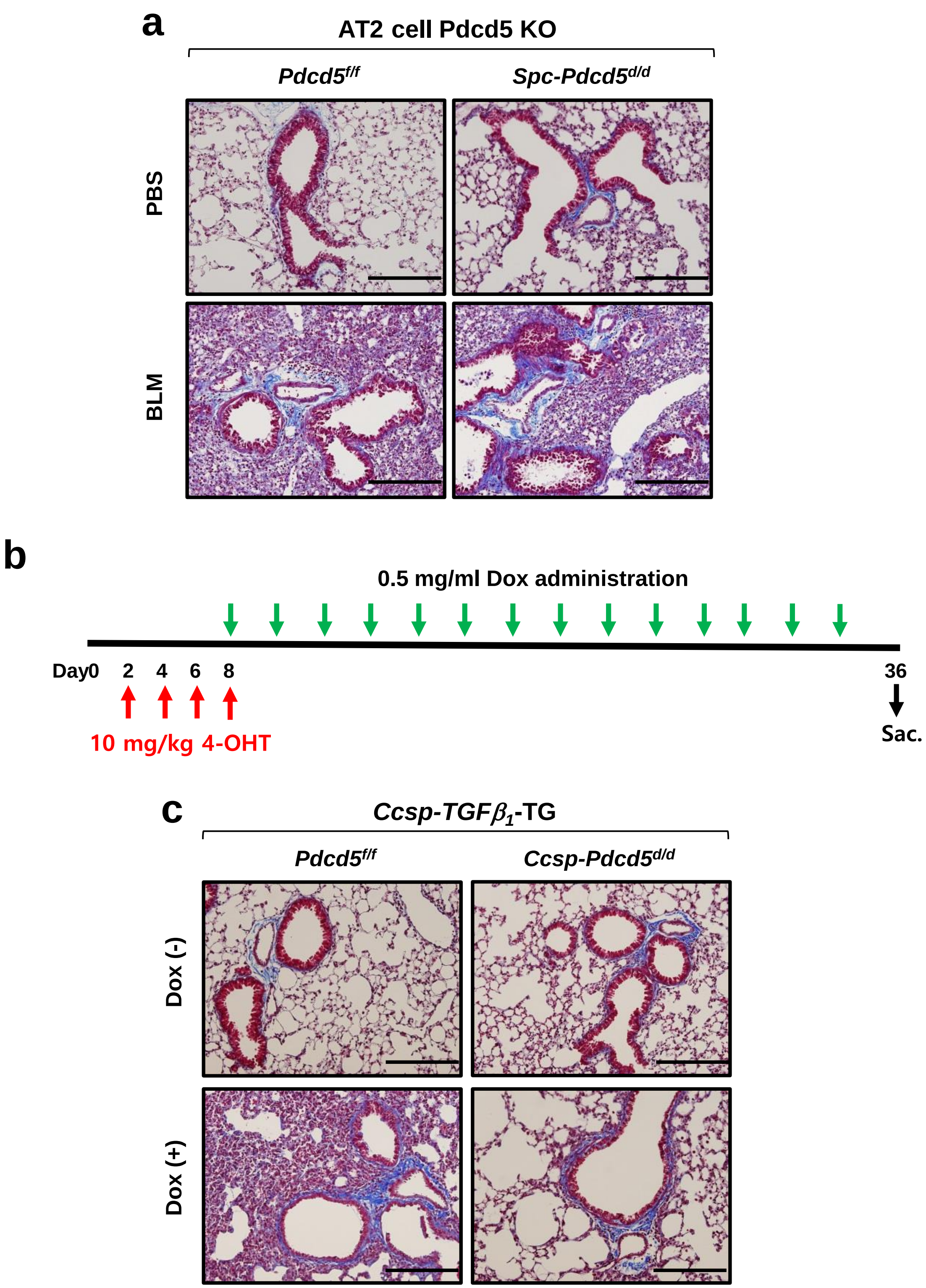

Supplementary Fig. 5. Deletion of *Pdcd5* in club cells, but not AT2 cells, prevents lung fibrosis. a MTS staining illustrates fibrotic regions in the lungs of *Pdcd5*$^{f/f}$ and *Spc-Pdcd5*$^{d/d}$ mice after BLM injection. Scale bars = 200 μm **b** This experimental scheme was used to generate ablation of *Pdcd5* and overexpression of TGF-β_1 in the club cells. 4-OHT was injected 4 times every 2 days prior to Dox administration for 28 days. **c** MTS staining illustrates fibrotic regions in the lungs of *Ccsp-TGF*β_1-TG with *Pdcd5*$^{f/f}$ or *Ccsp-Pdcd5*$^{d/d}$ mice after Dox administration. Scale bars = 200 μm. Representative images for three mice from each group are shown.

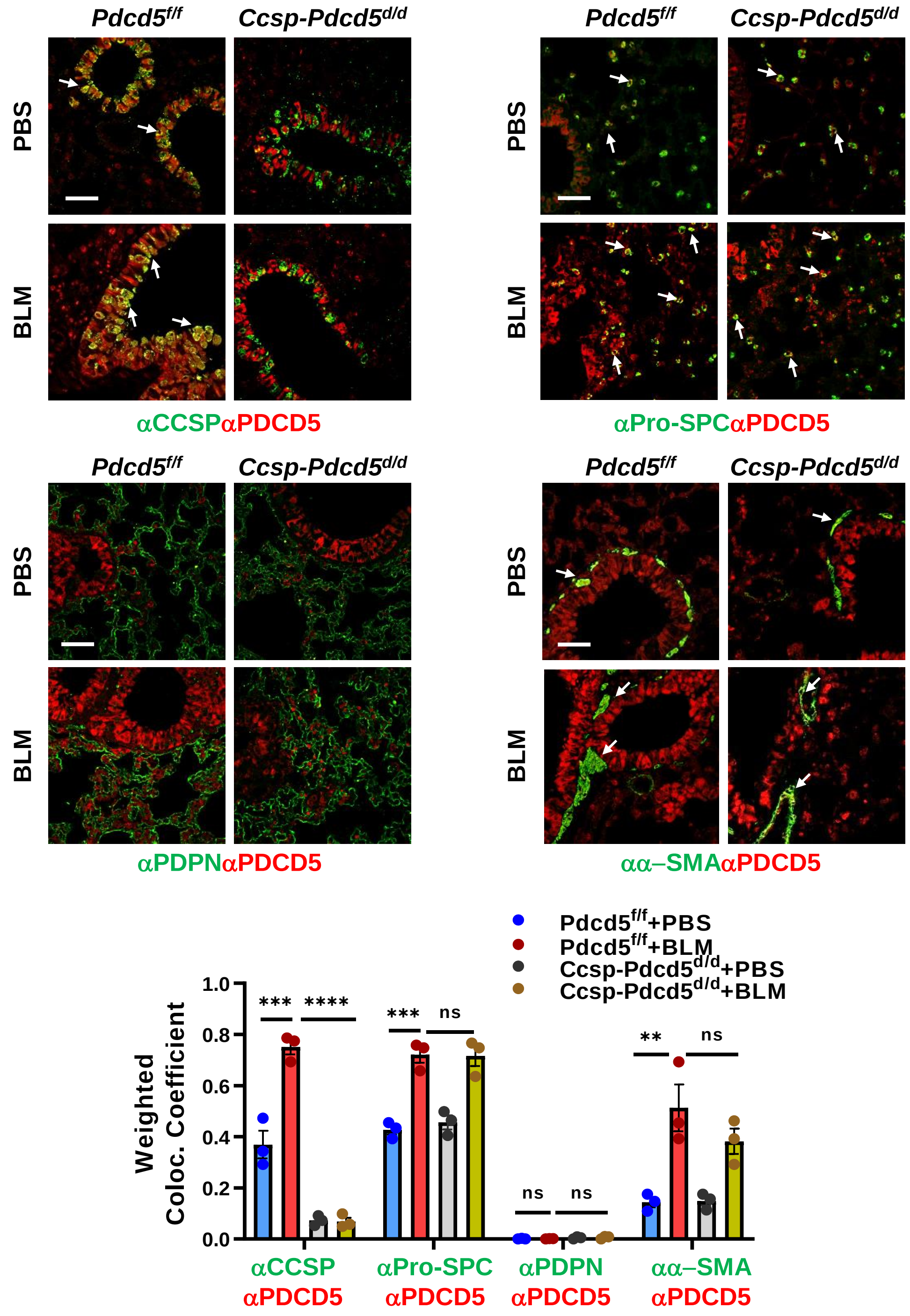

Supplementary Fig. 6. The effect of Club cell specific *Pdcd5* deletion on induction of PDCD5 expression by BLM. Co-immunofluorescence analysis of PDCD5 and indicated antibodies was carried out on lung tissues from *Pdcd5*$^{f/f}$ and *Ccsp-Pdcd5*$^{d/d}$ mice (Scale bars = 50 μm). White arrows indicate co-localization regions. Weighted co-localized coefficient was calculated using ZEN 3.0 software. Error bars represent mean ± s.e.m. (n = 3 mice in each group); **p = 0.0055; ***p < 0.0006; ****p < 0.0001; ns, not significant, one-way ANOVA with Tukey's test. Source data are provided in the Source Data file.

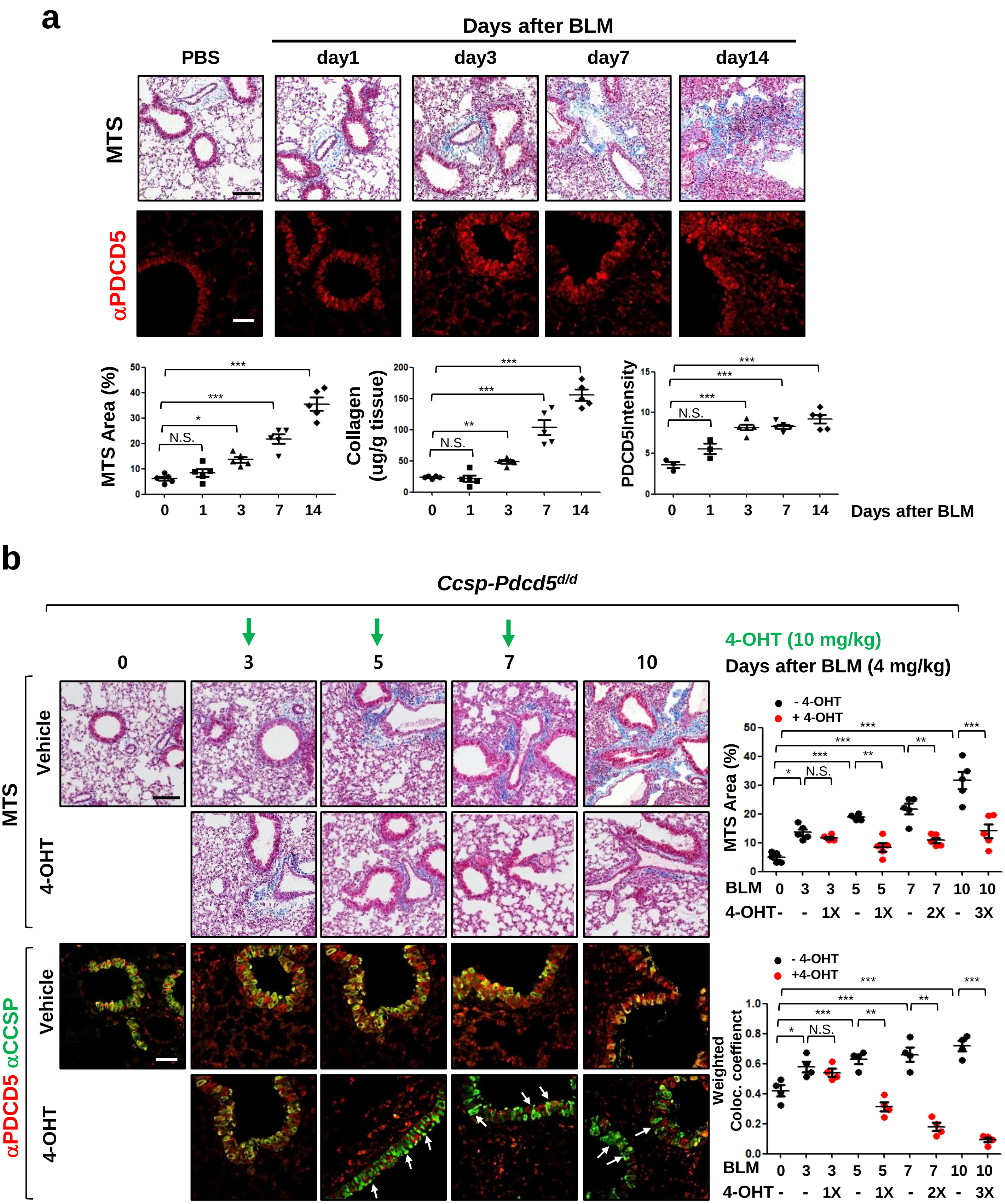

Supplementary Fig. 7. See next page for legend

Supplementary Fig. 7. The lung fibrosis following BLM injection was significantly suppressed by *Pdcd5* deletion. a In wild type mice, MTS and soluble collagen assay were conducted after the indicated days of BLM injection (4 mg/kg). Representative MTS images for five mice from each group are shown. Scale bar = 100 µm. Immunofluorescence analysis of PDCD5 was performed in the lungs. Red florescence intensities were quantified by image J software. Scale bars = 50 µm. Error bars represent mean ± s.e.m. (n = 5 mice in each group except day 1 and 3 groups of PDCD5 intensity, n = 3); *p = 0.0177; ***p < 0.001; ns, not significant, one-way ANOVA followed by Tukey's test. **b** *Ccsp-Pdcd5*$^{d/d}$ mice were treated 4-OHT (10 mg/kg) from 2 days after BLM injection, and then mice were sacrificed at the indicated time point. MTS staining revealed the fibrotic regions in the lungs. Scale bar = 100 µm. Co-immunofluorescence analysis of PDCD5 (Red) and club cell-specific marker (CCSP, Green) was performed to verify deletion of *Pdcd5*. White arrows indicate diminished co-localization regions of PDCD5 and CCSP. Scale bars = 50 µm (left panel). Collagen fiber areas in MTS images were quantified using ImageJ software. Weighted co-localized coefficient was calculated using ZEN software. Error bars represent mean ± s.e.m. (n = 4 mice/group); *p < 0.0425; **p < 0.0044; ***p < 0.001; ns, not significant. (right panel). Statistical analysis was performed with one-way ANOVA with Tukey’s post hoc test. Source data are provided in the Source Data file.

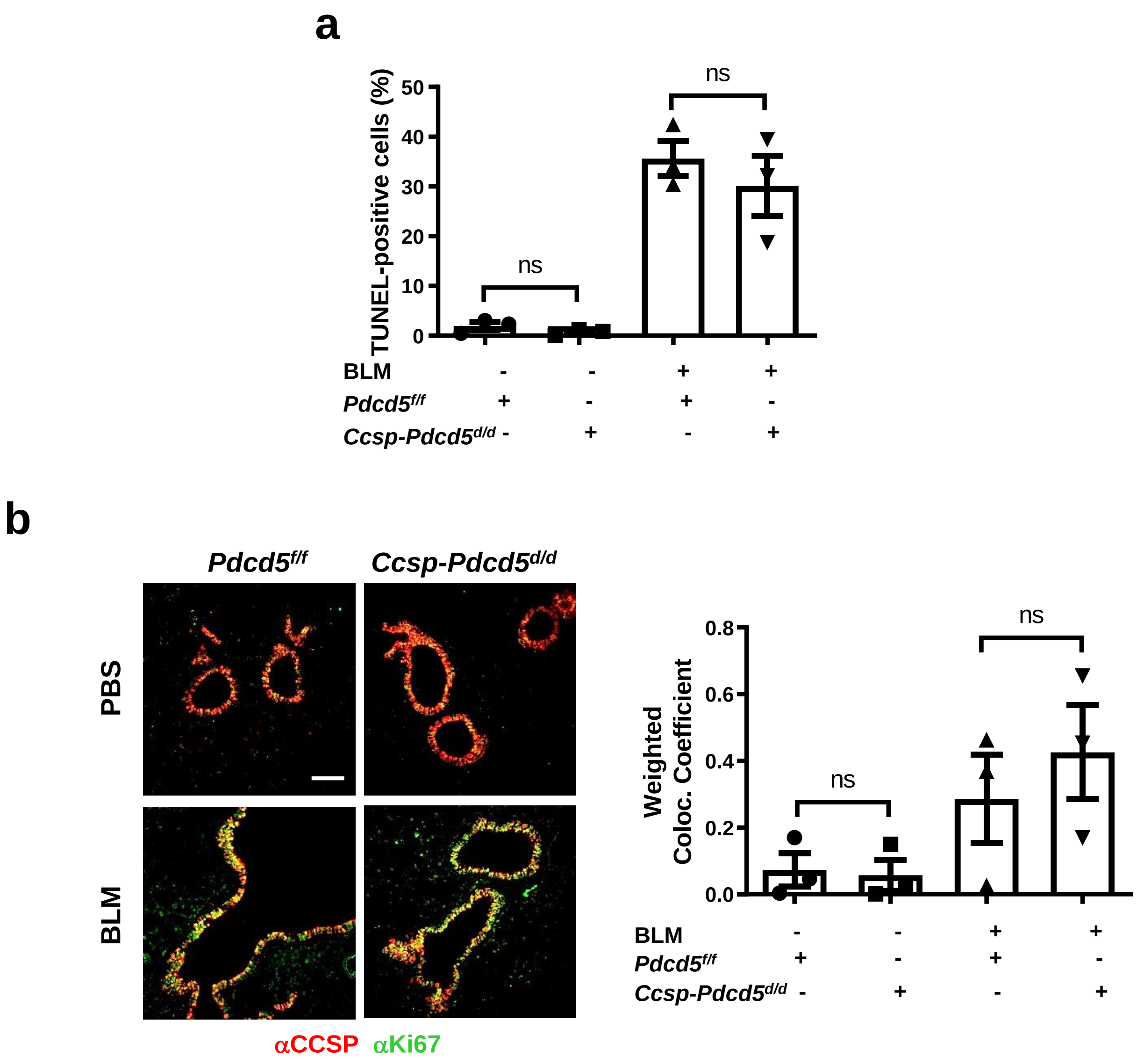

Supplementary Fig. 8. Deletion of *Pdcd5* in club cells had no effects on bleomycin-induced cell death and proliferation. a TUNEL assay was performed in lung tissue samples from BLM-induced *Pdcd5*$^{f/f}$ and *Ccsp-Pdcd5*$^{d/d}$ mice. TUNEL-positive cells were quantified using ImageJ software. Error bars represent mean ± s.e.m. (n = 3/group); ns, not significant. **b** CCSP and Ki67 double staining shows BLM-induced proliferation in the airway region in *Pdcd5*$^{f/f}$ and *Ccsp-Pdcd5*$^{d/d}$ mice. Scale bar = 100 µm. Weighted co-localized coefficients were calculated using ZEN 3.0 software. Error bars, mean ± s.e.m. (n = 3/group); ns, not significant. Statistical analysis was performed with one-way ANOVA with Tukey's post hoc test. Source data are provided in the Source Data file.

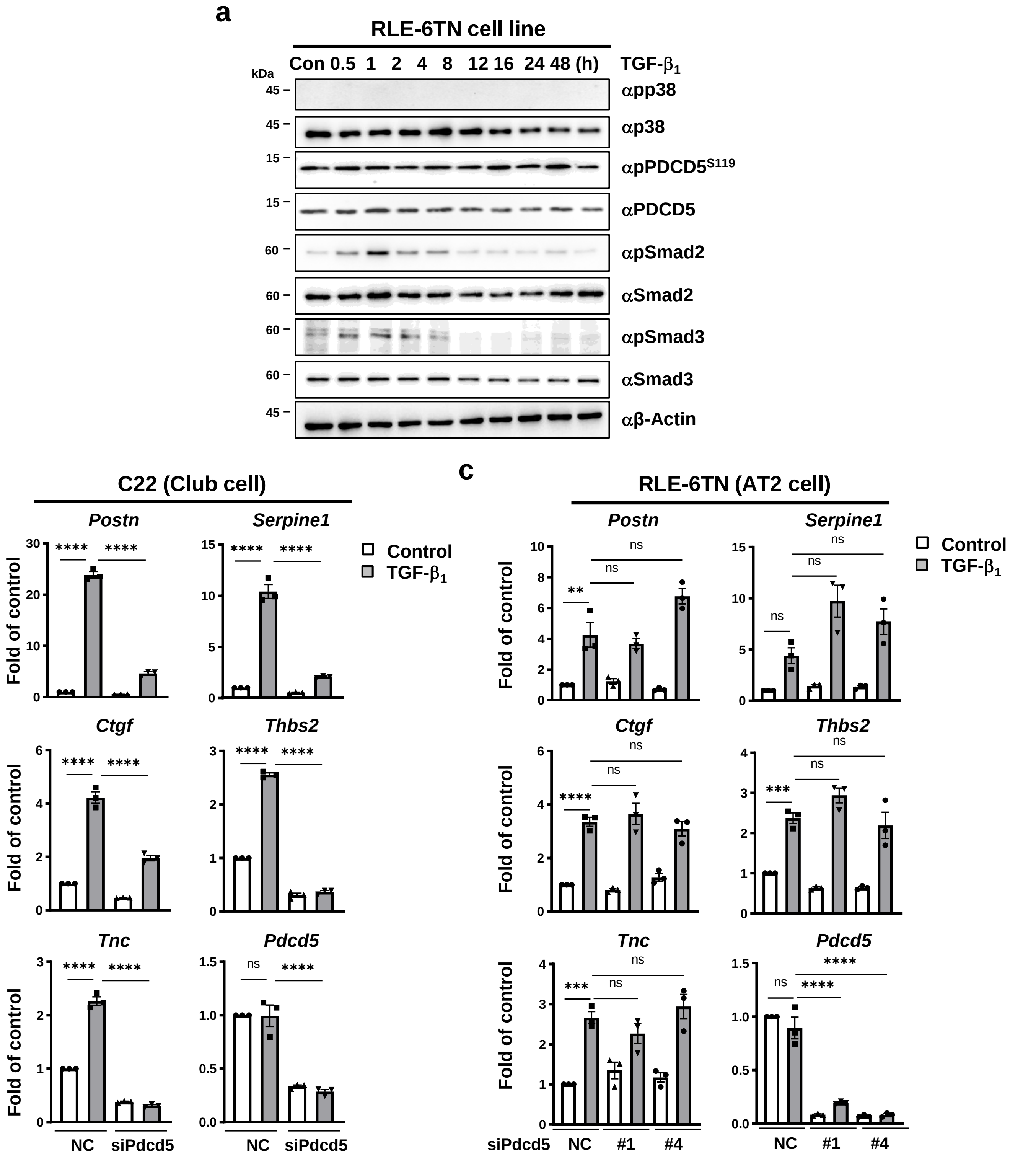

Supplementary Fig. 9. PDCD5 selectively regulates transcription of pro-fibrotic matricellular genes in the club cells but not AT2 cells. a RLE-6TN cells were treated with TGF-β_1 for the indicated time points. Whole-cell lysates were analyzed by immunoblotting using the indicated antibodies. Representative blots from three independent experiments are shown. **b** C22 cells were transfected with *Pdcd5* siRNAs for 24 hours and then treated with TGF-β_1 for 24 hours. The levels of the indicated genes were analyzed by real-time PCR. Error bars represent mean ± s.e.m. (n = 3/group); ****p < 0.0001; ns, not significant. **c** RLE-6TN cells were transfected with *Pdcd5* siRNAs for 36 hours and then with TGF-β_1 for 24 hours. The levels of indicated genes were analyzed by real-time PCR. Error bars represent mean ± s.e.m. (n = 3/group); **p = 0.0012; ***p < 0.0008; ****p < 0.0001; ns, not significant. Statistical analysis was performed with one-way ANOVA with Tukey's post hoc test (**a, b**). Source data are provided in the Source Data file.

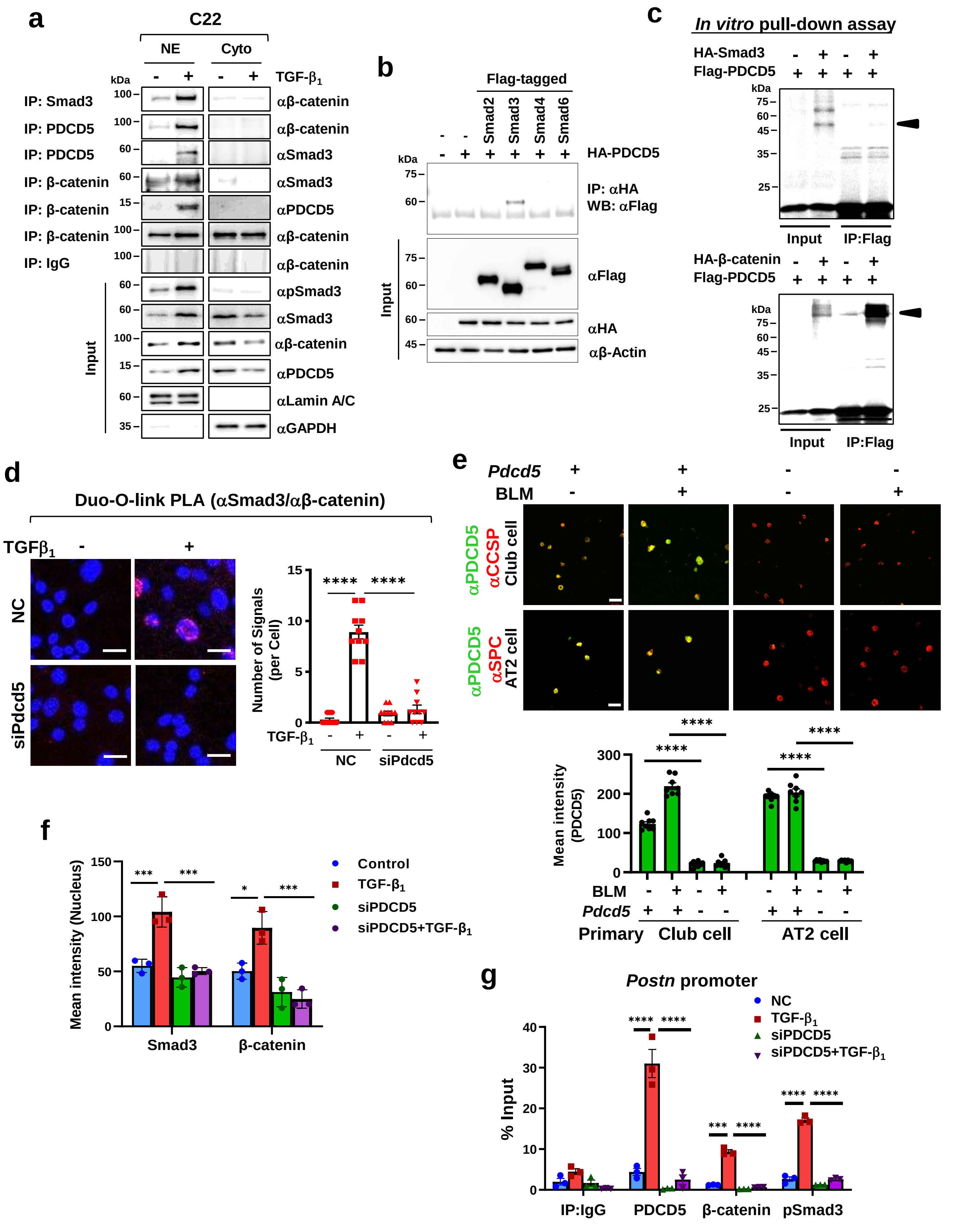

Supplementary Fig. 10. See next page for legend

Supplementary Fig. 10. PDCD5 mediates formation of the PDCD5/β-catenin/Smad3 complex in response to TGF-b. a C22 cells were treated with TGF-β_1 (20 ng/ml) for 12 hours. Following cell fractionation, nuclear or cytosolic fraction was immunoprecipitated and analyzed by immunoblotting with the indicated antibodies. **b** C22 cells were transfected with indicated DNA plasmids. Whole cell lysates were immunoprecipitated with an anti-HA antibody and immunoblotted with an anti-Flag antibody. **c** *In vitro* co-translated in rabbit reticulocyte with Flag-PDCD5 and HA-Smad3 or HA-β-catenin was immunoprecipitated with an anti-Flag antibody. Bound proteins were eluted and analyzed by autoradiography. **d** C22 cells were treated with TGF-β_1 and the Smad3/β-catenin interaction was analyzed by *in situ* PLA analysis. Scale bars = 50 µm. The number of PLA-positive signals were quantified by ImageJ software. Error bars represent mean ± s.e.m. (n = 10/group), ****$p < 0.001$. **e** Primary club cells were isolated from *Pdcd5*$^{f/f}$ and *Ccsp-Pdcd5*$^{d/d}$ mouse lungs, and AT2 cells were isolated from *Pdcd5*$^{f/f}$ and *Spc-Pdcd5*$^{d/d}$ mice after BLM injection. Double staining against PDCD5/CCSP for primary club cells, and PDCD5/SPC for AT2 cells. Scale bars = 20 µm. Mean intensity of PDCD5 was calculated using ZEN 3.0 software. Error bars represent mean ± s.e.m. (n = 10/group), ****$p < 0.001$. **f** Quantification of nuclear intensity shown in Fig. 3f, using ZEN 3.0 software**.** Error bars represent mean ± s.e.m. (n = 3/group), *$p = 0.0123$; *** $p < 0.0006$. **g** ChIP assay was performed with indicated antibodies. Precipitated samples were analyzed by real-time PCR, and results are presented as the percentage of input. Error bars represent mean ± s.e.m. (n = 3/group), **** $p < 0.001$. Statistical analysis was performed with one-way ANOVA with Tukey's post hoc test (**e, f, g**). Representative blots and images from three independent experiments are shown (**a-e**). Source data are provided in the Source Data file

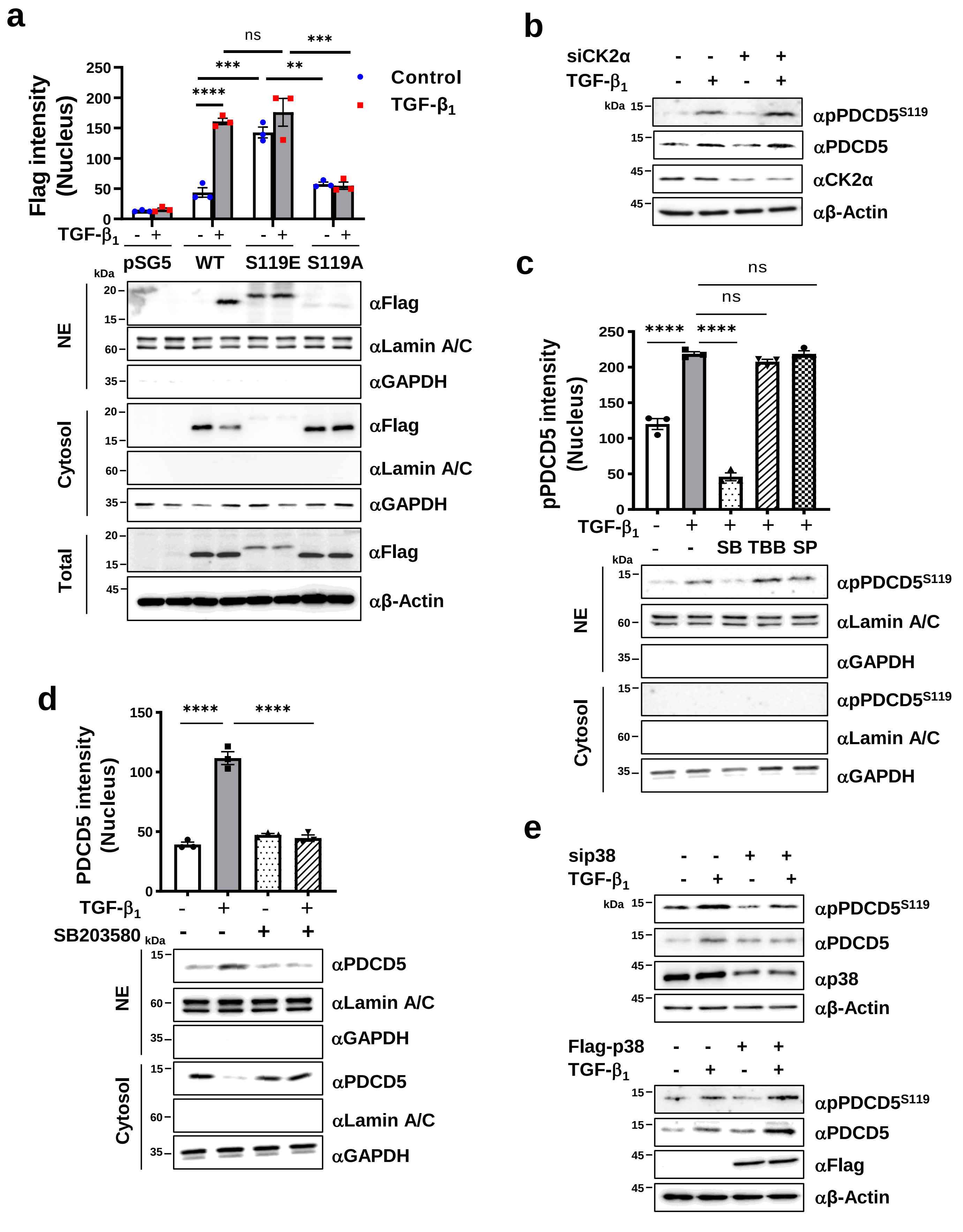

Supplementary Fig. 11. See next page for legend

Supplementary Fig. 11. p38 MAPK, not CK2, mediates the phosphorylation and stabilization of PDCD5 in the club cells. a, C22 cells were transfected with the indicated plasmids. Nuclear Flag intensity of Fig. 4a was analyzed using ZEN 3.0 software. Error bars represent mean ± s.e.m. (n = 3/group), $^{**}p = 0.0016$; $^{***}p < 0.0004$; $^{****}p < 0.0001$; ns, not significant. The nuclear and cytosolic fractions were analyzed by immunoblotting. Total lysates were immunoblotted with the indicated antibodies. **b** C22 cells were transfected with CK2α siRNA and then treated with 20 ng/ml TGF-β_1 for 1 hour. Immunoblotting was performed using the indicated antibodies. **c, d** Nuclear intensity of the IF staining shown in Fig. 4c (**c**) and Fig. 4e (**d**). Error bars represent mean ± s.e.m. (n = 3/group), $^{****}p < 0.0001$; ns, not significant. Nuclear and cytosolic fractions were analyzed by immunoblotting, using the indicated antibodies. Statistical analysis was performed with one-way ANOVA with Tukey's post hoc test (**a, c, d**). **e** C22 cells were transfected with p38 siRNA, or Flag-p38-pcDNA3.0 overexpression vector and then treated with 20 ng/ml TGF-β_1 for 1 hour. Immunoblotting was performed using the indicated antibodies. Representative blots from three independent experiments are shown (**a-e**). Source data are provided in the Source Data file

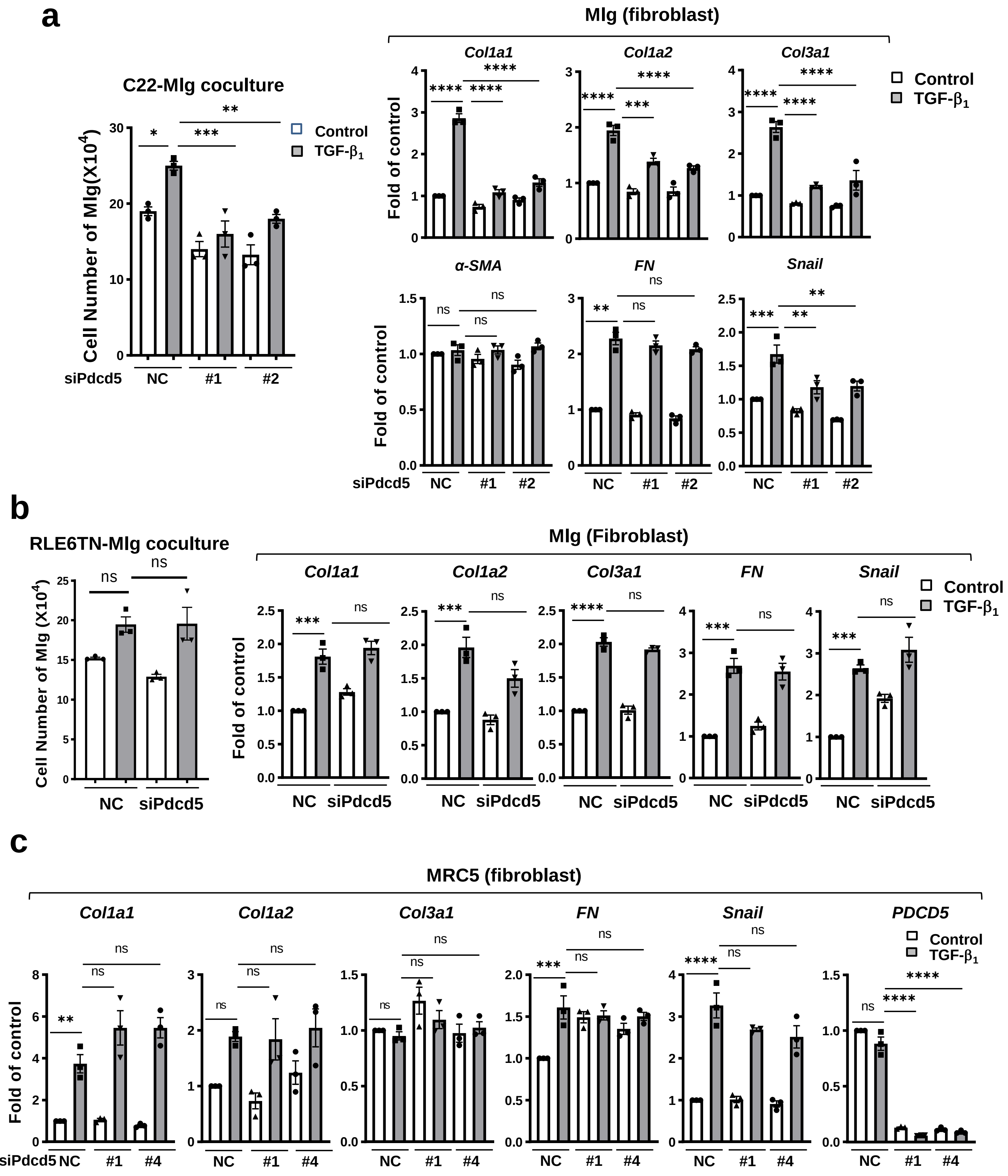

Supplementary Fig. 12. See next page for legend

Supplementary Fig. 12. PDCD5 promotes fibroblast activation in a club cell-specific manner. a Transwells plated with C22 cells were transfected with Pdcd5 siRNAs for 36 hours. Mlg mouse lung fibroblasts were plated on 24-well plate one day before TGF-β_1 treatment. Transwells were then transferred to the Mlg plates. After 24 hours TGF-β_1 (20 ng/ml) treatment, fibroblast cells were counted using an automated cell counter (left panel). Mlg fibroblast cells were cultured in C22 CM and levels of indicated genes were analyzed by quantitative RT-PCR. Error bars represent mean ± s.e.m. (n = 3/group), *$p < 0.0237$; **$p < 0.0055$; *** $p < 0.0007$; **** $p < 0.0001$; ns, not significant. **b** RLE-6TN cells plated on Transwells were transfected with Pdcd5 siRNAs for 36 hours. Mlg cells were plated on 24-well plate one day before TGF-β_1 treatment. Transwells were then transferred to the Mlg plates. After 24 hours of TGF-β_1 treatment, Mlg fibroblast cells were counted using an automated cell counter (left panel). Mlg fibroblast cells were cultured in C22 CM and levels of indicated genes were analyzed by qRT-PCR. Error bars represent mean ± s.e.m. (n = 3/group), **$p < 0.0069$; ***$p < 0.009$; ****$p < 0.0001$; ns, not significant. **c** MRC5 cells, human lung fibroblast cell line, were transfected with siPdcd5 for 36 hours and treated with 20 ng/ml TGF-β_1 for 24 hours. The levels of indicated genes were analyzed by qRT-PCR. Error bars, mean ± s.e.m. (n = 3/group), *$p = 0.0257$; **$p < 0.0074$; ***$p < 0.0001$; ns, not significant. Statistical analysis was performed with one-way ANOVA with Tukey's post hoc test. Source data are provided in the Source Data file

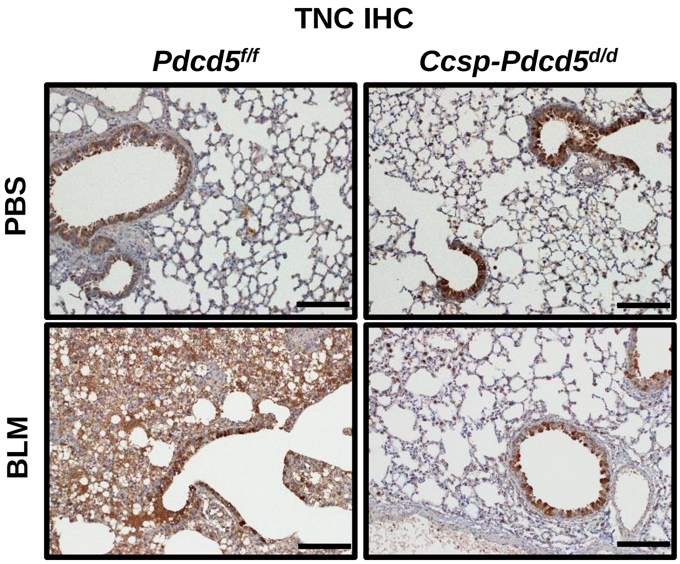

Supplementary Fig. 13. Ablation of *Pdcd5* significantly diminishes secretion of TNC in the lung. IHC with TNC antibody was performed in lung tissue from BLM-induced *Pdcd5*$^{f/f}$ and *Ccsp-Pdcd5*$^{d/d}$ mice. Scale bars = 100 μm. Representative images for three mice from each group are shown.

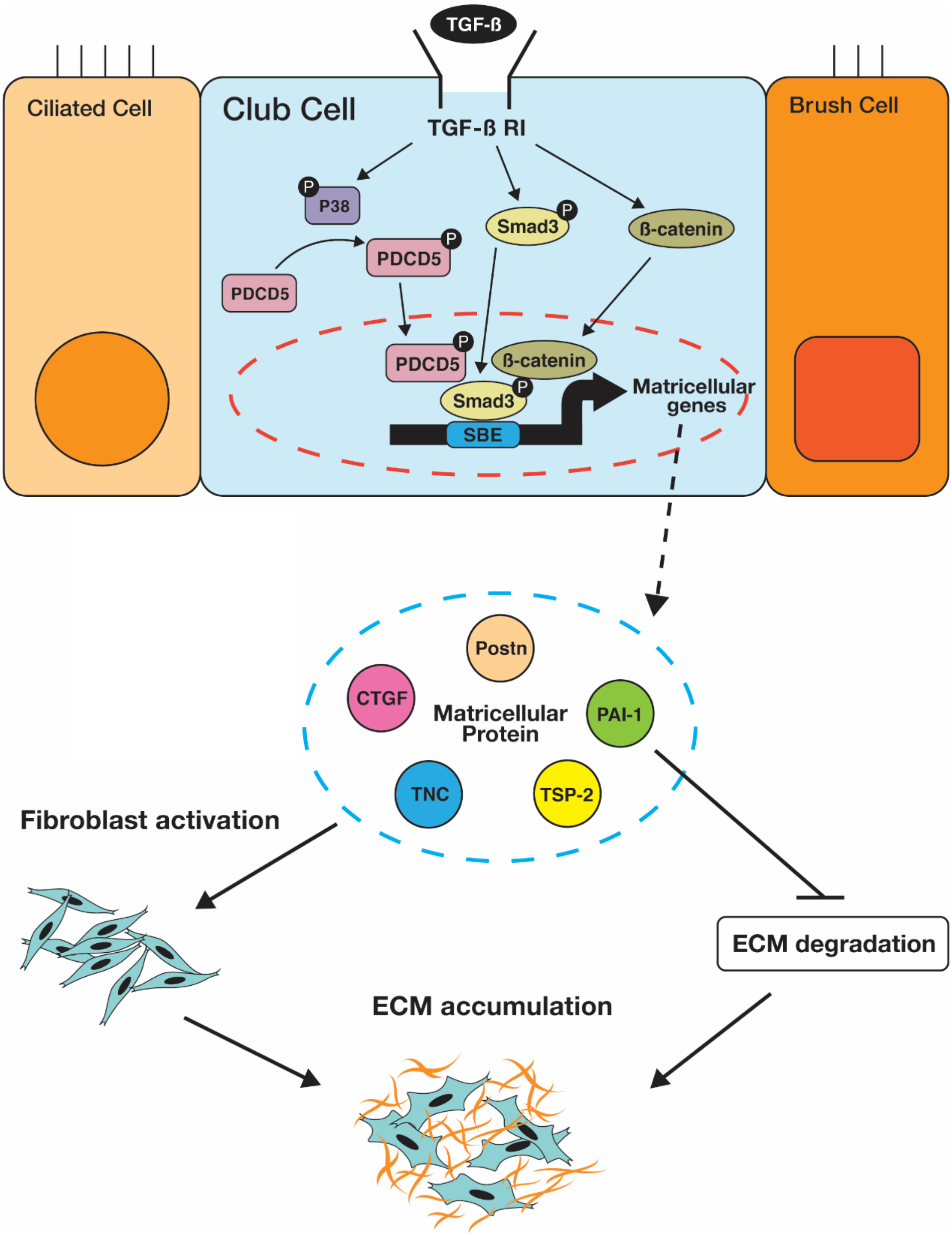

Supplementary Fig. 14. Schematic model of study findings. Upon TGF-β_1 stimuli, Ser-119 of PDCD5 is phosphorylated by p38 MAPK. Phosphorylated PDCD5 is translocated into the nucleus and interacts with the β-catenin/Smad3 complex to mediate transcriptional activation of matricellular genes in club cells. Secreted matricellular proteins from club cells activate fibroblasts, which leads to increased collagen accumulation.
