# Supplementary material for: Club cell-specific role of programmed cell death 5 in pulmonary fibrosis
Source: Nat Commun. 2021 May 19;12:2923. doi: 10.1038/s41467-021-23277-8 (PMC8134485; doi:10.1038/s41467-021-23277-8)
Supplement: Supplementary file 2 — Reporting Summary [file 41467_2021_23277_MOESM2_ESM.pdf]

nature research

Corresponding author(s): Ho-Geun Yoon, Myung Hyun Sohn

Last updated by author(s): Mar 22, 2021

Reporting Summary

Nature Research wishes to improve the reproducibility of the work that we publish. This form provides structure for consistency and transparency in reporting. For further information on Nature Research policies, see Authors & Referees and the Editorial Policy Checklist .

Statistics

For all statistical analyses, confirm that the following items are present in the figure legend, table legend, main text, or Methods section.

n/a	Confirmed	
☐	☒	The exact sample size (*n*) for each experimental group/condition, given as a discrete number and unit of measurement
☐	☒	A statement on whether measurements were taken from distinct samples or whether the same sample was measured repeatedly
☐	☒	The statistical test(s) used AND whether they are one- or two-sided<br>*Only common tests should be described solely by name; describe more complex techniques in the Methods section.*
☒	☐	A description of all covariates tested
☐	☒	A description of any assumptions or corrections, such as tests of normality and adjustment for multiple comparisons
☐	☒	A full description of the statistical parameters including central tendency (e.g. means) or other basic estimates (e.g. regression coefficient) AND variation (e.g. standard deviation) or associated estimates of uncertainty (e.g. confidence intervals)
☐	☒	For null hypothesis testing, the test statistic (e.g. *F*, *t*, *r*) with confidence intervals, effect sizes, degrees of freedom and *P* value noted<br>*Give P values as exact values whenever suitable.*
☒	☐	For Bayesian analysis, information on the choice of priors and Markov chain Monte Carlo settings
☒	☐	For hierarchical and complex designs, identification of the appropriate level for tests and full reporting of outcomes
☒	☐	Estimates of effect sizes (e.g. Cohen's *d*, Pearson's *r*), indicating how they were calculated

Our web collection on statistics for biologists contains articles on many of the points above.

Software and code

Policy information about availability of computer code

Data collection	Imaging: ZEN software (Black edition, v3.0, Carl Zeiss)<br>FACS sorting: BD FACSDiva software (v8, BD Biosciences)
Data analysis	ImageJ Fiji 1.52i and ZEN software (v3.0) were used for image data analysis.<br>Prism Software (GraphPad v9) and R (v3.3.2) used to analyze the statistics.<br>Bowtie 2 (v2.3.4.3), Bedtools (v2.26), Gene set enrichment analysis (GSEA, v4.0.1, Broad institute), Molecular Signatures Database (MSigDB v7.0), DAVID (v6.8, http://david.abcc.ncifcrg.gov/)and R (v4.0.3) were used for RNA sequencing data analysis.<br>Sorting data was analyzed using FlowJo software (v10, TreeStar Inc.).

For manuscripts utilizing custom algorithms or software that are central to the research but not yet described in published literature, software must be made available to editors/reviewers. We strongly encourage code deposition in a community repository (e.g. GitHub). See the Nature Research guidelines for submitting code & software for further information.

Data

Policy information about availability of data

All manuscripts must include a data availability statement. This statement should provide the following information, where applicable:

- Accession codes, unique identifiers, or web links for publicly available datasets
- A list of figures that have associated raw data
- A description of any restrictions on data availability

All data supporting the findings of this study are available within the paper and its supplementary information files. RNA-seq data (Fig. 3b) have been deposited in the NCBI Gene Expression Omnibus (GEO), Accession No.GSE143841.
Source data for figures shown in this study are available upon request if not available in Supplementary Data in the attached source data file. The source data underlying Figs.1b-d, f, g, 2b-e, 3a, c-e, g, 4b, d, f, 5a-c and Supplementary Figs 1b, 2a-d, 3a-b, 4e-f, 6-12 are provided as a Source data file.

Field-specific reporting

Please select the one below that is the best fit for your research. If you are not sure, read the appropriate sections before making your selection.

- [x] Life sciences
- [ ] Behavioural & social sciences
- [ ] Ecological, evolutionary & environmental sciences

For a reference copy of the document with all sections, see nature.com/documents/nr-reporting-summary-flat.pdf

Life sciences study design

All studies must disclose on these points even when the disclosure is negative.

Sample size	No statistical method was used to pre-select the sample size. The sample size was determined based on similar previous studies of our labratory and on previous experiments using similar methodologies. Detailed sample size were described in the figure legends.
Data exclusions	No data were excluded from the analyses.
Replication	All experiments were performed with at least 3 replicates. All attempts at replication were successful.
Randomization	For all animal experiments, aged-matched male mice were randomly allocated from the appropriate genotype.
Blinding	Blinding was not possible as the same investigators performed experiments and analyzed the data

Reporting for specific materials, systems and methods

We require information from authors about some types of materials, experimental systems and methods used in many studies. Here, indicate whether each material, system or method listed is relevant to your study. If you are not sure if a list item applies to your research, read the appropriate section before selecting a response.

Materials & experimental systems

n/a	Involved in the study	
☐	☒	Antibodies
☐	☒	Eukaryotic cell lines
☒	☐	Palaeontology
☐	☒	Animals and other organisms
☐	☒	Human research participants
☒	☐	Clinical data

Methods

n/a	Involved in the study	
☒	☐	ChIP-seq
☐	☒	Flow cytometry
☒	☐	MRI-based neuroimaging

Antibodies

Antibodies used

Immunohistochemistry:
anti-PDCD5 (Proteintech, 12456-1-AP, various lots including 00065223, 1:100)
anti-CTGF (Abcam, ab6992, lot GR2047-102, 1:100),
anti-Periostin (Abcam, ab14041, lot GR3237481-7, 1:100)
anti-TNC (Abcam, ab108930, lot GR320921-7, 1:100)
normal rabbit IgG (Santacruz, sc2027, lot G2516, 1:100)

Immunofluorescence imaging:
anti-PDCD5 (R&D Systems, clone MAB7325, various lots including CGAK 0219021 ,1:100)
anti-p-PDCD5-Ser119 (manufactured from Ab frontier, 1:200)
anti-Flag (Sigma-Aldrich, F1804, clone AC15, lot 079M4799v, 1:500),
anti-CCSP (MerckMillpore, 07-623, clone 2982296, 1:100)
anti-CCSP (santacruz, sc9773, clone S-20, 1:100)
anti-pro-SP-C (Santacruz, sc-13979, clone FL-197, lot F2016, 1:100)
anti-PDPN (R&D Systems, AF3244, lot WUF 0319121, 1:100)
anti-Muc5AC (ThermoFisher, MA5-12178, 1:100)
anti-Foxj1 (ThermoFisher, 14-9965-82, 1:100)
anti-Scgb3a2 (R&D Systems, AF3545, 1:100),
anti-alpha-SMA (abcam, ab5694, lot GR3263275-15 1:200)
anti-Ki-67 (abcam, ab833, 1:200)
DyLight 488 anti-mouse IgG (Vectorlab, DI-2488, lot Z1030, 1:500)
DyLight 488 anti-rabbit IgG (Vectorlab, DI-1488, ZA0122, 1:500)

DyLight 549 anti-rabbit IgG (Vectorlab, DI-1549, lot W0914, 1:500)
DyLight 549 anti-mouse IgG (Vectorlab, DI-2549, lot W0830, 1:500)

Immunoprecipitation, immunoblot, and ChIP assay:
anti-PDCD5 (R&D Systems, MAB7325, various lots including 00065223, 1:100 for IP, 1:1000 for IB, 2ug for ChIP)
anti-p-PDCD5-Ser119 (manufactured from Ab frontier, 1:500 for IB)
anti-CTGF (Abcam, ab6992, lot GR2047-102, 1:100 for IB)
anti-Periostin (Abcam, ab14041, lot GR3237481-7, 1:1000 for IB)
anti-TNC (Abcam, ab108930, lot GR320921-7, 1:1000 for IB)
anti-TSP-2 (Abcam, ab84469, lot GR3353171-1, 1:1000 for IB)
anti-Smad2 (Cell Signaling, 5339, clone D43B4, 1:1000 for IB)
anti-Smad3 (Cell Signaling, 9513, lot 2, 1:100 for IP, 1:1000 for IB),
anti-p-Smad2 (Cell Signaling, 3101, clone 138D4, 1:1000 for IB),
anti-p-Smad3 (Cell Signaling, 9520, clone C25A9, 1:100 for IP, 1:1000 for IB, 1ug for ChIP),
Lamin A/C (Cell Signaling, 4777, clone 4C11, 1:2000 for IB)
anti-Flag (Sigma-Aldrich, F1804, clone AC15, lot 079M4799v, 1:5000 for IB)
anti-HA (Santacruz, sc-805, clone Y-11, lot K2015, 1:200 for IP)
anti-GAPDH (Santa Cruz, sc-32233, 1:1000 for IB),
anti-β-catenin (BD Biosciences, 610153, 1:100 for IP, 1:1000 for IB, 1ug for ChIP),
anti- β-actin (Sigma-Aldrich, A5441, 1:5000 for IB),
anti-p-p38 (Cell signaling, 4511, clone D3F9, 1:1000 for IB)
anti-p38 (Cell signaling, 8690, clone D13E1, 1:1000 for IB)

Primary lung cell sorting:
Alexa488 anti-CCSP (MerckMillpore, 07-623, 1:100),
FITC anti-CD74 (BD Biosciences, 555318, clone In-1, 1:100),
APC anti-EpCAM (ThermoFisher, 17-5791-82, clone G8.8, 1:100),
Rat IgG2a K Isotype control FITC (eBioscience, 11-4321, 1:100),
Rat IgG2a K Isotype Control APC (eBioscience, 17-4321, 1:100)

Validation

All antibodies are commercially available and validated by the manufacturer, except phospho-PDCD5 ser119 antibody.
Validation statements are available from manufacturer's website
anti-PDCD5 (https://www.ptglab.com/products/PDCD5-Antibody-12456-1-AP.htm)
anti-CTGF (https://www.abcam.com/ctgf-antibody-ab6992.html)
anti-Periostin (https://www.abcam.com/periostin-antibody-ab14041.html)
anti-TNC (https://www.abcam.com/tenascin-c-antibody-epr4219-ab108930.html)
normal rabbit IgG (https://www.scbt.com/ko/p/normal-mouse-igg)
anti-PDCD5 (https://www.rndsystems.com/products/human-mouse-rat-pdcd5-antibody-728906_mab7325)
anti-Flag (https://www.sigmaaldrich.com/catalog/product/sigma/f1804?lang=ko&region=KR),
anti-CCSP (https://www.merckmillipore.com/KR/ko/product/Anti-Clara-Cell-Secretory-Protein-Antibody,MM_NF-07-623)
anti-CCSP (https://www.scbt.com/ko/p/cc10-antibody-s-20)
anti-pro-SP-C (https://www.scbt.com/p/sp-c-antibody-fl-197?requestFrom=search)
anti-PDPN (https://www.rndsystems.com/products/mouse-podoplanin-antibody_af3244)
anti-Muc5AC (https://www.thermofisher.com/antibody/product/MUC5AC-Antibody-clone-45M1-Monoclonal/MA5-12178)
anti-Foxj1 (https://www.thermofisher.com/antibody/product/FOXJ1-Antibody-clone-2A5-Monoclonal/14-9965-82)
anti-Scgb3a2 (https://www.rndsystems.com/products/human-ugrp1-scgb3a2-antibody_af3545),
anti-alpha-SMA (https://www.abcam.com/alpha-smooth-muscle-actin-antibody-ab5694.html)
anti-Ki-67 (https://www.abcam.com/ki67-antibody-ab833.html)
DyLight 488 anti-mouse IgG (https://vectorlabs.com/dylight-488-horse-anti-mouse-igg-antibody.html)
DyLight 488 anti-rabbit IgG (https://vectorlabs.com/dylight-488-goat-anti-rabbit-igg-antibody.html)
DyLight 549 anti-rabbit IgG (https://vectorlabs.com/dylight-549-goat-anti-rabbit-igg-antibody.html)
DyLight 549 anti-mouse IgG (https://vectorlabs.com/dylight-549-horse-anti-mouse-igg-antibody.html)
anti-PDCD5 (https://www.rndsystems.com/products/human-mouse-rat-pdcd5-antibody-728906_mab7325)
anti-CTGF (https://www.abcam.com/ctgf-antibody-ab6992.html)
anti-Periostin (https://www.abcam.com/periostin-antibody-ab14041.html)
anti-TNC (https://www.abcam.com/tenascin-c-antibody-epr4219-ab108930.html)
anti-TSP-2 (https://www.abcam.com/thrombospondin-2-antibody-ab84469.html)
anti-Smad2 (https://www.cellsignal.com/products/primary-antibodies/smad2-d43b4-xp-rabbit-mab/5339)
anti-Smad3 (https://www.cellsignal.com/products/primary-antibodies/smad3-antibody/9513),
anti-p-Smad2 (https://www.cellsignal.com/products/primary-antibodies/phospho-smad2-ser465-467-antibody/3101),
anti-p-Smad3 (https://www.cellsignal.com/products/primary-antibodies/phospho-smad3-ser423-425-c25a9-rabbit-mab/9520),
Lamin A/C (https://www.cellsignal.com/products/primary-antibodies/lamin-a-c-4c11-mouse-mab/4777)
anti-Flag (https://www.sigmaaldrich.com/catalog/product/sigma/f1804?lang=ko&region=KR)
anti-HA (https://www.scbt.com/ko/p/ha-probe-antibody-y-11)

anti-GAPDH (https://www.scbt.com/ko/p/gapdh-antibody-6c5?requestFrom=search),
anti-β-catenin (https://www.bdbiosciences.com/us/applications/research/stem-cell-research/cancer-research/human/purified-mouse-anti--catenin-14beta-catenin/p/610153),
anti- β-actin (https://www.sigmaaldrich.com/catalog/product/sigma/a5441?lang=ko&region=KR),
anti-p-p38 (https://www.cellsignal.com/products/primary-antibodies/phospho-p38-mapk-thr180-tyr182-d3f9-xp-rabbit-mab/4511)
anti-p38 (https://www.cellsignal.com/products/primary-antibodies/p38-mapk-d13e1-xp-rabbit-mab/8690)
Alexa488 anti-CCSP (https://www.merckmillipore.com/KR/ko/product/Anti-Clara-Cell-Secretory-Protein-Antibody,MM_NF-07-623),
FITC anti-CD74 (https://www.bdbiosciences.com/eu/applications/research/intracellular-flow/intracellular-antibodies-and-isotype-controls/anti-mouse-antibodies/fitc-rat-anti-mouse-cd74-in-1/p/555318),
APC anti-EpCAM (https://www.thermofisher.com/antibody/product/CD326-EpCAM-Antibody-clone-G8-8-Monoclonal/17-5791-82),
Rat IgG2a K Isotype control FITC (https://www.thermofisher.com/antibody/product/Rat-IgG2a-kappa-clone-eBR2a-Isotype-Control/11-4321-80),
Rat IgG2a K Isotype Control APC (https://www.thermofisher.com/antibody/product/Rat-IgG2a-kappa-clone-eBR2a-Isotype-Control/17-4321-81)

Anti-phospho-PDCD5 ser-119 antibody was validated by western blot using PDCD5 S119A mutant construct (Nat commun. 2015 Jun 16;6:7390, Supplementary Fig.10b).

Eukaryotic cell lines

Policy information about cell lines

Cell line source(s)	C22 cell line was purchased from Sigma-aldrich, and primary mouse fibroblast were purchased from Cell Biologics (Chicago, IL, USA). RLE-6TN cell line were presented by Prof. Sang Myun Park in Ajou University, Korea (purchased from ATCC, Exp Mol Med. 2012 31;44(5):319-29). Mlg and MRC5 cell line were obtained from Korean cell line bank (Seoul, Korea).
Authentication	None of these cell lines were authenticated by us, but displayed expected morphology.
Mycoplasma contamination	Cells were tested for mycoplasma contamination and were negative.
Commonly misidentified lines (See ICLAC register)	There is no commonly misidentified cell lines were used in the study.

Animals and other organisms

Policy information about studies involving animals; ARRIVE guidelines recommended for reporting animal research

Laboratory animals	Male wildtype C57/BL6, Pdcd5fl/fl X Scgb1a1CreER, Pdcd5fl/fl X SftpcCreER, TGF-beta1 transgenic, ROSAmTmG reporter mice (mean age 8 weeks).
Wild animals	The study did not involve wild animals.
Field-collected samples	The study did not involve samples collected from the field.
Ethics oversight	All animal experiments were approved by the Institutional Animal Care and Use Committee of Yonsei University College of Medicine (IACUC no. 2015-0047).

Note that full information on the approval of the study protocol must also be provided in the manuscript.

Human research participants

Policy information about studies involving human research participants

Population characteristics	Patient clinical characteristics are summarized in the Table S1.
Recruitment	All patient and control materials were obtained from the tissue bank at Severance Hospital (Seoul, Korea). Written informed consent was obtained from all subjects. There was no self-selection bias involved.
Ethics oversight	This study was approved by the institutional review board of Severance Hospital (protocol no. 4-2016-0453). Informed consent was obtained from all the patients.

Note that full information on the approval of the study protocol must also be provided in the manuscript.

Flow Cytometry

Plots

Confirm that:

☒ The axis labels state the marker and fluorochrome used (e.g. CD4-FITC).

☒ The axis scales are clearly visible. Include numbers along axes only for bottom left plot of group (a 'group' is an analysis of identical markers).

☒ All plots are contour plots with outliers or pseudocolor plots.

☒ A numerical value for number of cells or percentage (with statistics) is provided.

Methodology

Sample preparation	Mouse lungs were dissociated using a mouse lung dissociation kit (Miltenyi Biotec, #130-095-927) with gentleMACSTM Dissociator (Miltenyi Biotec). After removing red blood cells by lysis buffer (Miltenyi Biotec), dissociated lung cells were stained with antibodies described above.
Instrument	BD FACSAriaTM III cell sorter (BD Biosciences)
Software	FlowJo software V10 (TreeStar Inc.)
Cell population abundance	Mouse lung club cell (CCSP+EpCAM+) and AT2 cell (CD74+EpCAM+) were sorted based on the gating strategy.
Gating strategy	Club cells: FSC-A/SSC-A -> SSC-W/SSC-H (sigle cell) -> FSC-W/FSC-H (single cell)-> CCSP+EpCAM+<br>AT2 cells: FSC-A/SSC-A-> SSC-W/SSC-H (sigle cell) -> FSC-W/FSC-H (single cell)-> sigle cell -> CD74+EpCAM+

☒ Tick this box to confirm that a figure exemplifying the gating strategy is provided in the Supplementary Information.
